# Hydroxylation of diverse flavonoids by CYP450 BM3 variants: biosynthesis of eriodictyol from naringenin in whole cells and its biological activities

**DOI:** 10.1186/s12934-016-0533-4

**Published:** 2016-08-05

**Authors:** Luan Luong Chu, Ramesh Prasad Pandey, Narae Jung, Hye Jin Jung, Eun-Hee Kim, Jae Kyung Sohng

**Affiliations:** 1 Department of Life Science and Biochemical Engineering, SunMoon University, 70 Sunmoon-ro 221, Tangjeong-myeon, Asan-si, Chungnam 31460 South Korea; 2 Department of BT-Convergent Pharmaceutical Engineering, SunMoon University, 70 Sunmoon-ro 221, Tangjeong-myeon, Asan-si, Chungnam 31460 South Korea; 3 Division of Magnetic Resonance, Korea Basic Science Institute, Ochang, Chungbuk 363-883 South Korea

**Keywords:** Cytochrome P450 BM3, Monooxygenase, Hydroxylation, Flavonoids

## Abstract

**Background:**

Cytochrome P450 monooxygenase constitutes a significant group of oxidative enzymes that can introduce an oxygen atom in a high regio- and stereo-selectivity mode. We used the *Bacillus megaterium* cytochrome P450 BM3 (CYP450 BM3) and its variants namely mutant 13 (M13) and mutant 15 (M15) for the hydroxylation of diverse class of flavonoids.

**Results:**

Among 20 flavonoids, maximum seven flavonoids were hydroxylated by the variants while none of these molecules were accepted by CYP450 BM3 in in vitro reaction. Moreover, M13 exhibited higher conversion of substrates than M15 and CYP450 BM3 enzymes. We found that M13 carried out regiospecific 3ʹ-hydroxylation reaction of naringenin with the highest conversion among all the tested flavonoids. The apparent *K*_*m*_ and *k*_*cat*_ values of M13 for naringenin were 446 µM and 1.955 s^−1^, respectively. In whole-cell biotransformation experiment with 100 µM of naringenin in M9 minimal medium with 2 % glucose in shake flask culture, M13 showed 2.14- and 13.96-folds higher conversion yield in comparison with M15 (16.11 %) and wild type (2.47 %). The yield of eriodictyol was 46.95 µM [~40.7 mg (13.5 mg/L)] in a 3-L volume lab scale fermentor at 48 h in the same medium exhibiting approximately 49.81 % conversion of the substrate. In addition, eriodictyol exhibited higher antibacterial and anticancer potential than naringenin, flavanone and hesperetin.

**Conclusions:**

We elucidated that eriodictyol being produced from naringenin using recombinant CYP450 BM3 and its variants from *B. megaterium*, which shows an approach for the production of important hydroxylated compounds of various polyphenols that may span pharmaceutical industries.

**Electronic supplementary material:**

The online version of this article (doi:10.1186/s12934-016-0533-4) contains supplementary material, which is available to authorized users.

## Background

Cytochrome P450 enzymes (CYP450s) catalyze diverse types of chemical reactions such as hydroxylation, epoxidation, reduction, oxidation, deamination, peroxidation, *O*-demethylation, desulfonation and dehalogenation [1, 2]. In general, CYP450s introduce an atom of oxygen into a substrate and the concomitant reduction of the second oxygen atom to water. CYP450s mediated reactions offer great potential for the realistic application of regio-selective and stereo-selective hydroxylation of diverse substrates [3–7]. CYP450 BM3 (CYP102A1) from *Bacillus megaterium* is a self-sufficient fatty acid monooxygenase, which has been studied since last 30 years [8] and has emerged as a potent biocatalyst for biotechnological application [9]. CYP450 BM3 is a class II P450 enzyme that consists of natural fusion between heme-Fe-dependent monooxygenase domain and the electron transfer flavin mononucleotide (FMN)/flavin adenine dinucleotide (FAD) reductase domain in a single continuous 119**-**kDa polypeptide. The natural substrates of CYP450 BM3 are C_12_–C_20_ fatty acids that are hydroxylated at very high activity at sub-terminal position [10]. Moreover, through rational design or directed evolution, protein engineering of CYP450 BM3 has been carried out to expand the substrates flexibility to generate pharmaceutically important molecules [11–15]. These recent advances suggest that CYP450 BM3 mutant (M13: R47L/L86I/F87V/L188Q; M15: R47L/E64G/F87V/E143G/L188Q/E267V) can be developed as a biocatalyst for drug discovery and synthesis. However, there have been no reports of either CYP450 BM3 wild type or mutant M13 and M15 modifying flavonoid groups of compounds to produce diverse hydroxylated products.

Flavonoids are one of the most numerous and structurally diverse natural products present in the plant kingdom [16]. They are known to have multi-beneficial medicinal and chemo-preventive activities in human health. Flavonoids have been shown to act as antioxidant [17], antibacterial [18], anti-inflammatory [19], hepato-protective [20], and anticancer properties [21]. However, the pharmaceutical application of these compounds is limited, because of their low water solubility and instability. Hydroxylation of the activated or non-activated carbon atoms in the flavonoids improves their metabolic stability and enhances the solubility, which greatly enhances their biological properties [22]. Some of the hydroxylated flavonoids exhibited better antioxidants than their parental flavonoids [23], suppression of ultraviolet (UV)-B induced skin cancer [24] and modulates multidrug resistance transporters and induces apoptosis [25]. Naringenin, a typical flavanone that is also known as (2*S*)-naringenin or 4′,5,7-trihydroxyflavanone, has a wide spectrum of pharmacological activities, including immunomodulatory and cellular antioxidant [26], anti-flammatory and anti-carcinogenic effects [27]. Moreover, it was also found to be beneficial for preventing the onset of chronic diseases like diabetes, obesity, and hypertension [28, 29]. *O*-Glycosylation and *O*-methylation are major modification reactions in enzymatic synthesis as well as biotransformation of naringenin [30–32]. But, the hydroxylation of flavonoids including naringenin has not been well studied. The recombinant *S. cerevisiae* cells overexpressing *PcCYP65a2* derived from the white-rot fungus *Phanerochaete chrysosporium* exhibited naringenin hydroxylation at 3′-position to yield eriodictyol [33]. Flavonoids hydroxylase from *Catharanthus roseus* [34] and *Gerbera hybrida* [35] have also been characterized; however these studies did not use them as biocatalysts, because of difficulty in enzyme expression in a heterologous system.

**Fig. 1:**
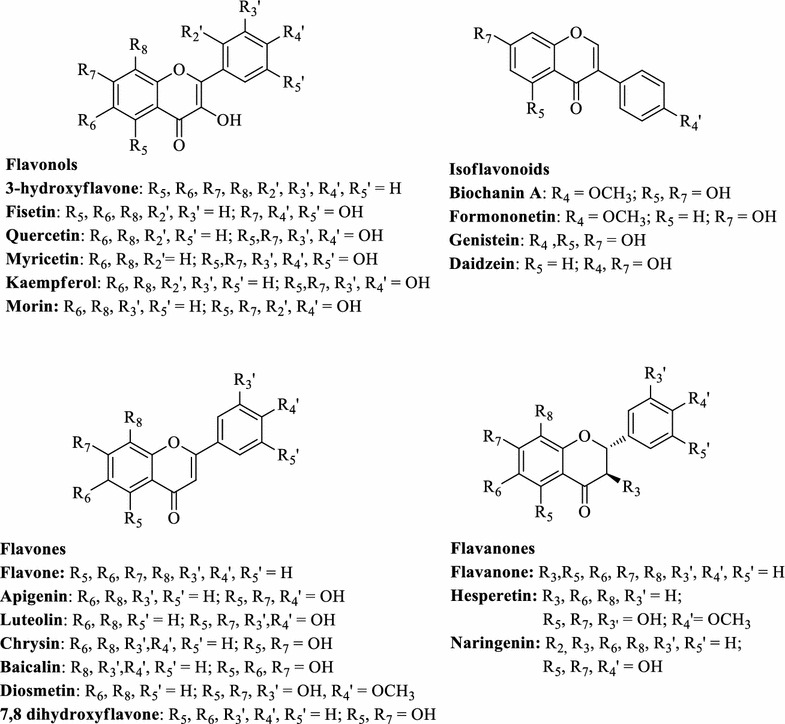
Structures of the flavonoids used in this study for in vitro reaction using CYP450 BM3 and its variants

In this study, we identified CYP450 BM3 variants capable of hydroxylating diverse sets of flavonoids tested (Fig. 1). We achieved regiospecific hydroxylation of flavonoids with high bioconversion of naringenin to eriodictyol by using one of the variants of CYP450 BM3, M13 when expressed in *Escherichia coli*. The product eriodictyol was tested for antibacterial activity against Gram-positive organism such as *Bacillus subtilis, Micrococcus luteus* and *Staphylococcus aureus*. Moreover, we compared the anticancer properties of the same molecule with naringenin, flavanone and hesperetin on AGS (gastric carcinoma), HCT116 (colon carcinoma), HepG2 (hepatic carcinoma) and HeLa (epithelioid cervix carcinoma) cancer cell lines.

## Results

### Expression, purification and spectral analysis of P450 BM3

**Fig. 2:**
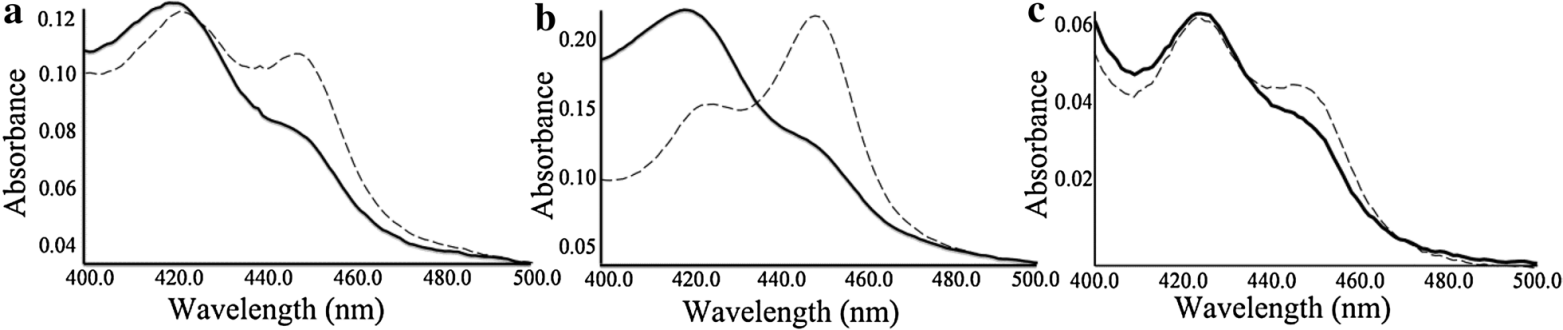
CO–binding spectra of protein **a** CYP450 BM3 wild type, **b** M13 and **c** M15. The *dotted line* denotes the oxidized form, and *solid line* denotes the reduced form

The sodium dodecyl sulfate polyacrylamide gel electrophoresis (SDS-PAGE) analysis of soluble and insoluble fraction of CYP450 BM3 and mutant variants M13 and M15 showed a protein band of approximate size of 119 kDa. Most of the protein was found in insoluble fraction. Yet, we have not tried to improve the amount of soluble protein using different growth conditions and isopropyl-β-D-thiogalactopyranoside (IPTG) concentrations. Simply, the soluble cytosolic fraction was collected using Amicon^®^Ultra-100 device—100,000 NMWL. The oxidized forms of the three proteins CYP450 BM3, M13 and M15 showed typical spectral properties of CYP enzymes with absorption at 420 nm. The sodium dithionite reduced spectra of the CYPs showed diminished absorption maxima in the Soret region. The peak maximum in the CO-difference spectra was at 450 nm. The Soret shift of proteins CYP450 BM3, M13 and M15 from 418 nm (Fig. 2a), 419 nm (Fig. 2b), and 420 nm (Fig. 2c) to 450 nm, respectively (for the Fe^II^-CO complex) are indicative of native Fe^II^-CO complexes of CYPs [36]. The expression yields of protein CYP450 BM3, M13 and M15 determined from the CO-difference spectra using an extinction molecular coefficient of *€*_450–490_ = 91 mM^−1^ cm^−1^ were 747.253, 1923.077, 472.527 nM/L, respectively. We determined the concentrations of proteins CYP450 BM3, M13 and M15 using the Bradford method to be approximately 4.705, 7.699 and 7.405 µg/mL, respectively.

### In vitro reaction

**Fig. 3:**
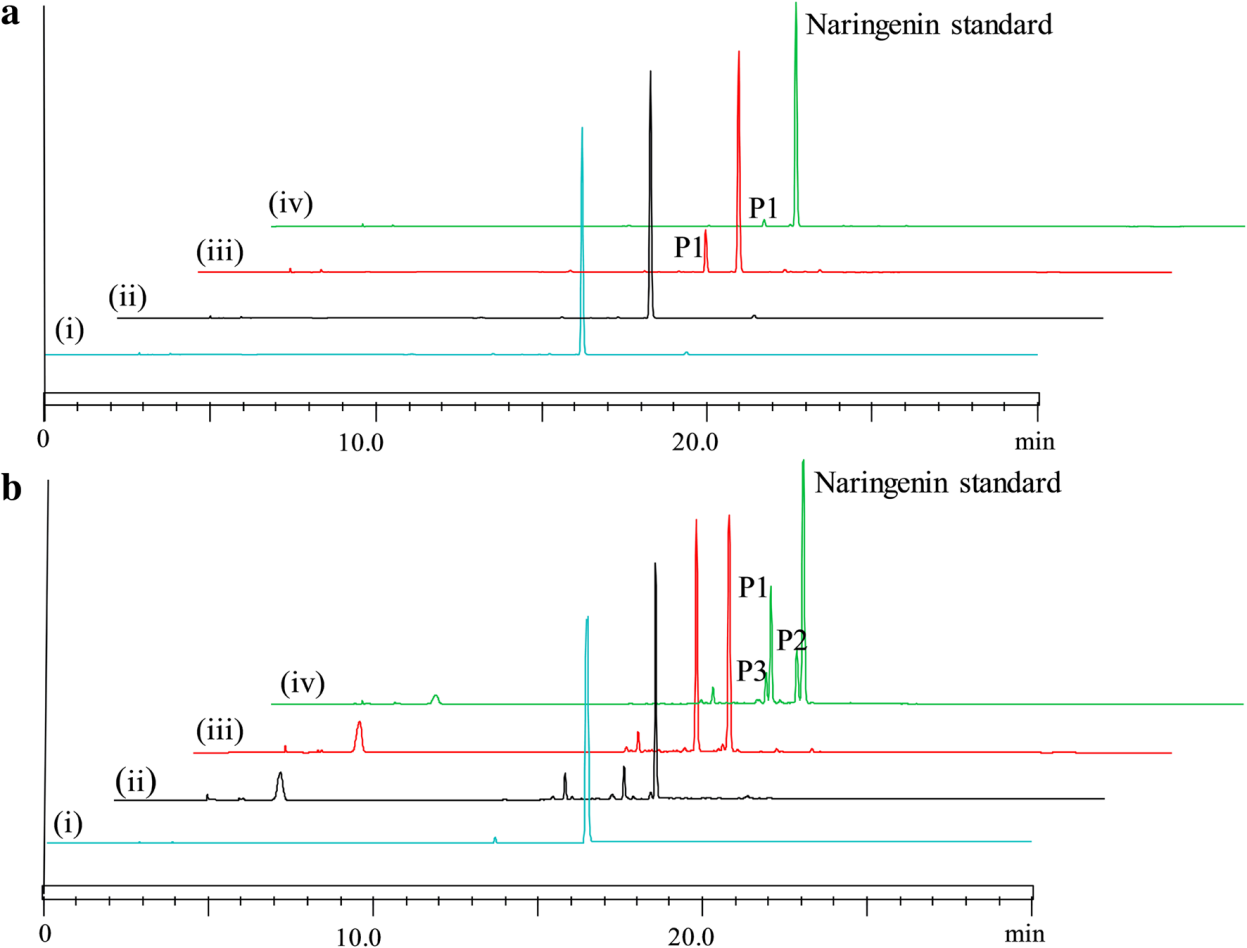
HPLC-PDA analysis of **a** in vitro hydroxylation reaction mixture and **b** whole cells bioconversion of naringenin. (*i*) control reaction of naringenin using *E. coli* BL21 (DE3); (*ii*) hydroxylation of naringenin with CYP450 BM3, (*iii*) M13 and (*iv*) M15, respectively

In vitro reaction of three proteins was carried out with twenty different flavonoids (flavonols, flavones, flavanones) and isoflavonoids under identical conditions as mentioned in methods. The reaction mixture was analyzed by high performance liquid chromatography-photodiode array (HPLC-PDA) for the preliminary analysis of hydroxylated products. Out of 20 substrates tested, seven flavonoids [naringenin, flavanone, genistein, daidzein, biochanin A, apigenin, 3-hydroxyflavone (3-HF)] were found to be hydroxylated with M13 and M15 mutant variants. We were unable to find catalytic activity of CYP450 BM3 with all of the flavonoids tested. The HPLC-PDA analysis also showed higher catalytic activity of M13 as a monooxygenase than M15. The comparative conversion percentage of each substrate to products with M13 and M15 are presented in Table 1. Each product of the reaction was characterized by UV absorbance maxima and high-resolution quadruple time-of-flight electrospray ionization-mass spectrometry (HR-QTOF ESI/MS) which is shown in Table 1 and supplementary files. Among flavonoids, the conversion of naringenin to hydroxylated derivative (retention time for peak 1 (*t*_*RP1*_) ~15.426 min, Fig. 3a(iii); calculated mass for molecular formula C_15_H_13_O_6_ for [M + H]^+^*m/z*^+^ ~ 289.0712 for which observed mass [M + H]^+^*m/z*^+^ ~ 289.0710, λ_max_: 287 nm) by protein M13 was 15.16 % in comparison with 2.82 % by protein M15 (Fig. 3a(iv); Additional file 1: Figure S1). Similarly, the conversion of apigenin to the product (*t*_*Ra1*_ ~ 15.274 min, calculated mass for molecular formula C_15_H_11_O_6_ for [M + H]^+^*m/z*^+^ ~ 287.0556 for which observed mass [M + H]^+^*m/z*^+^ ~ 287.0550, λ_max_: 247 and 339 nm) was 4.89 % by M13 variant which was only 1.14 % with M15 (Additional file 1: Figure S2). Interestingly, protein M15 did not exhibit any catalytic activity towards 3-HF, however, protein M13 catalyzed 3-HF to hydroxylated product (*t*_*Rh1*_ ~ 17.470 min, calculated mass for molecular formula C_15_H_11_O_4_ for [M + H]^+^*m/z*^+^ ~ 255.0657 for which observed mass [M + H]^+^*m/z*^+^ ~ 255.0734, λ_max_: 241, 306 and 339 nm) with 4.47 % conversion rate (Additional file 1: Figure S3). Furthermore, we were also able to detect two mono-hydroxylated flavanone (*t*_*Rf2*_ ~ 16.355 min, calculated mass for molecular formula C_15_H_13_O_3_ for [M + H]^+^*m/z*^+^ ~ 241.0865 for which observed mass [M + H]^+^*m/z*^+^ ~ 241.0864, λ_max_: 224 nm; and *t*_*Rf3*_ ~ 16.798 min, calculated mass for molecular formula C_15_H_13_O_3_ for [M + H]^+^*m/z*^+^ ~ 241.0865 for which observed mass [M + H]^+^*m/z*^+^ ~ 257.0857, λ_max_: 224 nm) as well as di-hydroxylated flavanone derivatives (*t*_*Rf1*_ ~ 11.461 min, calculated mass for molecular formula C_15_H_13_O_4_ for [M + H]^+^*m/z*^+^ ~ 257.0814 for which observed mass [M + H]^+^*m/z*^+^ ~ 257.0811, λ_max_: 240, 278 and 339 nm) by M13 with conversion rates of 12.26 %, 8.87 % and 9.65 %, respectively (Additional file 1: Figure S4). Likewise, the analysis of genistein reaction mixture showed two hydroxylated products (*t*_*Rg1*_ ~ 14.836 min, calculated mass for molecular formula C_15_H_11_O_6_ for [M + H]^+^*m/z*^+^ ~ 287.0556 for which observed mass [M + H]^+^*m/z*^+^ ~ 287.0552, λ_max_: 260 nm; *t*_*Rg2*_ ~ 15.485, calculated mass for molecular formula C_15_H_11_O_6_ for [M + H]^+^*m/z*^+^ ~ 287.0556 for which observed mass [M + H]^+^*m/z*^+^ ~ 287.0569, λ_max_: 268 nm) with both protein variants M13 and M15 (Additional file 1: Figure S5). We also found similar pattern of hydroxylation with another isoflavonoid molecule-daidzein. Both the enzymes produced two hydroxylated daidzein products having exactly similar retention time and mass spectra (*t*_*Rd1*_ ~ 13.683 min, calculated mass for molecular formula C_15_H_11_O_5_ for [M + H]^+^*m/z*^+^ ~ 271.0606 for which observed mass [M + H]^+^*m/z*^+^ ~ 271.0607, λ_max_: 254 nm; *t*_*Rd2*_ ~ 14.369, calculated mass for molecular formula C_15_H_11_O_5_ for [M + H]^+^*m/z*^+^ ~ 271.0606 for which observed mass [M + H]^+^*m/z*^+^ ~ 271.0603, λ_max_: 257 nm) (Additional file 1: Figure S6). However, in the case of biochanin A, M13 enzyme not only hydroxylated it (*t*_*Rb3*_ ~ 17.400; calculated mass for molecular formula C_16_H_13_O_6_ for [M + H]^+^*m/z*^+^ ~ 301.0712 for which observed mass [M + H]^+^*m/z*^+^ ~ 301.0711, λ_max_: 267 nm) but also demethylated (*t*_*Rb2*_ ~ 17.096, calculated mass for molecular formula C_15_H_11_O_5_ for [M + H]^+^*m/z*^+^ ~ 271.0606 for which observed mass [M + H]^+^*m/z*^+^ ~ 271.0600, λ_max_: 260 nm). Moreover, the demethylated derivative was further hydroxylated (*t*_*Rb1*_ ~ 16.376 min, calculated mass for molecular formula C_15_H_11_O_6_ for [M + H]^+^*m/z*^+^ ~ 287.0556 for which observed mass [M + H]^+^*m/z*^+^ ~ 287.0556, λ_max_: 268 nm). In contrast, M15 only demethylated biochanin A in trace amount (Additional file 1: Figure S7). In overall, these in vitro results showed that CYP450 BM3 variants preferred to catalyze over different classes of flavonoids and isoflavonoids. Among several flavonoids, we chose naringenin for further study using the variants of CYP450 BM3 because of its higher in vitro conversion rate and single reaction product.

**Table 1:** The conversion product, HPLC-PDA, HR-QTOF ESI/MS and UV maxima analyses of acceptor substrates in in vitro reaction using CYP450 BM3 and its variant proteins M13 and M15

Substrates				Products					
Name	HPLC (*t*_*R*_) min	Mass [M + H]^+^ *m/z* ^+^~	UV maxima (nm)	Name	% conversion M13	% conversion M15	HPLC (*t*_*R*_) min	Mass [M + H]^+^ *m/z* ^+^~	UV maxima (nm)
Naringenin	16.429	273.0756	286	Hydroxylated	15.16	2.82	15.426	289.0710	287
Apigenin	16.241	271.0608	254, 336	Hydroxylated (a1)	4.89	1.14	15.274	287.0540	247, 339
3-HF	21.310	239.0702	235, 305	Hydroxylated (h1)	4.47	Trace	17.470	255.0734	241, 306, 339
Flavanone	21.645	225.0916	252, 318	Hydroxylated (f2)	12.26	Trace	16.355	241.0864	224
				Hydroxylated (f3)	8.87	Trace	16.798	241.0857	224
				Di-hydroxylated (f1)	9.65	5.69	11.461	257.0811	240; 278, 339
Genistein	16.622	271.0608	260	Hydroxylated (g1)	33.38	1.95	14.836	287.0552	260
				Hydroxylated (g2)	2.77	1.02	15.485	287.0569	268
Daizein	15.264	255.0656	248	Hydroxylated (d1)	14.09	5.37	13.683	271.0607	254
				Hydroxylated (d2)	4.81	1.67	14.369	271.0603	257
Biochanin A	19.444	285.0762	260	Hydroxylated (b3)	27.73		17.400	301.0711	267
				De-methylated (b2)	3.44	1.11	17.096	271.0600	260
				De-methylated plus hydroxylated (b1)	2.47	Trace	16.376	287.0556	268

CYP450 BM3 has not shown activity under the test conditions

### Kinetic parameters of naringenin oxidation by P450 BM3

We used two protein variants M13 and M15 to measure kinetic parameters for the hydroxylation of naringenin. Protein M15 did not show any or appreciable activities to determine kinetic parameters. Only protein M13 showed significantly elevated *Km* and *K*_*cat*_ for the 3′-hydroxylation reaction with 446 µM and 1.955 s^−1^, respectively. This result indicated that M13 could be used for the bioconversion of naringenin into hydroxylated metabolite.

### Whole cell biotransformations of naringenin

**Fig. 4:**
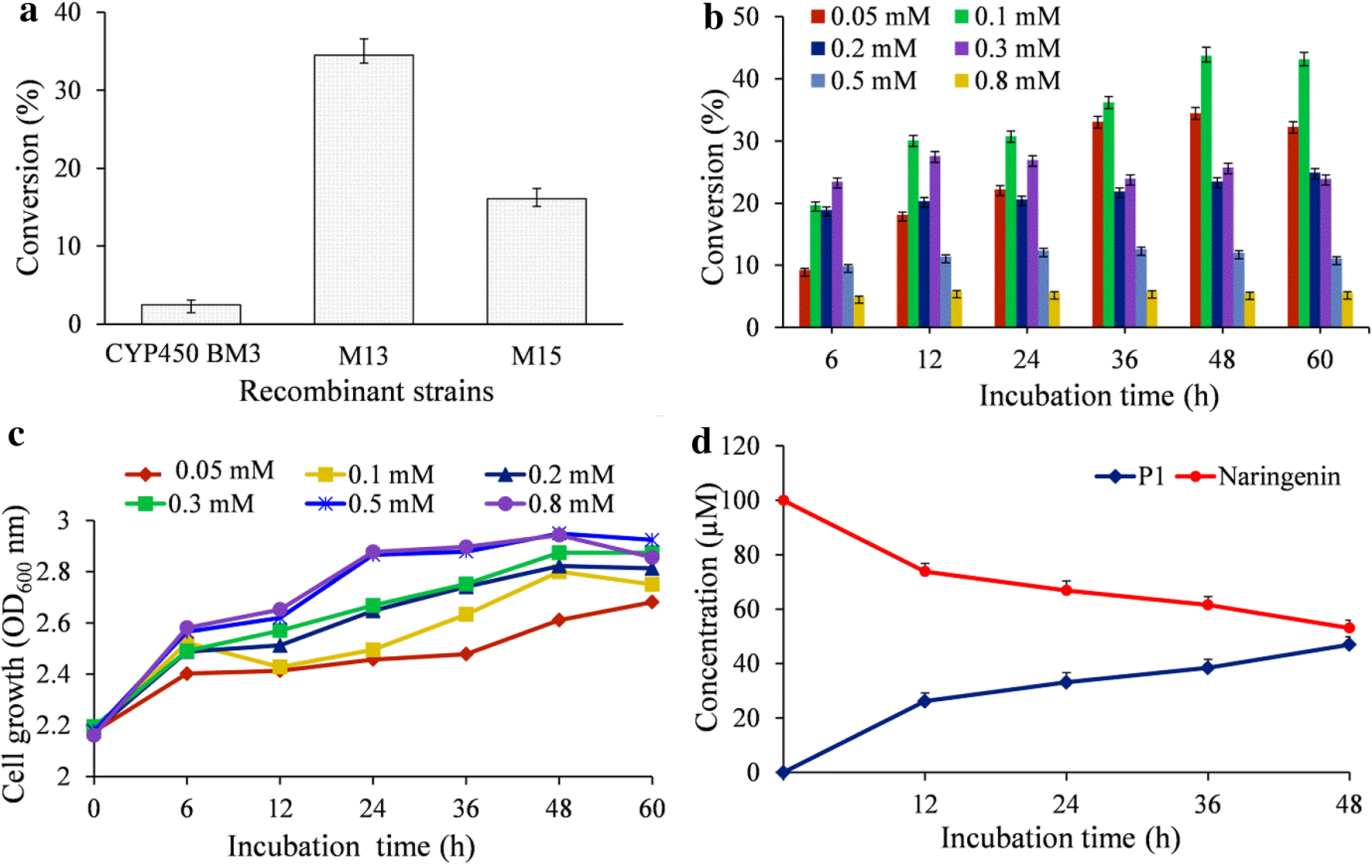
**a** Comparison of hydroxylated naringenin production from CYP450 BM3 wild type, M13 and M15 in M9 minimal medium supplemented with 2 % glucose at 48 h of incubation with 50 µM naringenin. **b** The substrate concentration optimization of naringenin (50–800 µM) in biotransformation. **c** Cell growth at OD_600_ nm while using various concentrations of naringenin during biotransformation. **d** The scale-up production of hydroxylated naringenin in 3-L fermentation at different time intervals. Standard deviations were calculated from the results of three independent experiments

We used the three recombinant strains harboring CYP450 BM3, M13 and M15 proteins expressing plasmids to check the bioconversion of exogenously supplemented naringenin as described in the “Methods” section. The HPLC-PDA chromatograms of extract from the biotransformation reaction of all three *E. coli* strains showed a peak at *t*_*R*_ ~ 15.426 min (P1), which could be the probable hydroxylated product of naringenin (*t*_*R*_ ~ 16.429 min) at the UV absorbance of 290 nm (Fig. 3b). These peaks were further analyzed by HR-QTOF ESI/MS. The exact mass of naringenin standard [M + H]^+^*m/z*^+^ was observed at ∼273.0756 corresponding to molecular formula C_15_H_13_O_5_ with λ_max_ ∼ 286 nm, for which the exact calculated mass was ∼273.0763 (Additional file 1: Figure S1). The mass spectra displayed the exact mass of hydroxylated product P1 [M + H]^+^*m/z*^+^ ∼ 289.0710 resemble molecular formula C_15_H_13_O_6_ with λ_max_ ∼ 287 nm, for with the exact calculated mass was ∼289.0712 (Additional file 1: Figure S1). Besides this product, M15 gene harboring strain had additional two new peaks at *t*_*R*_ ~ 16.241 min (P2) which has exact mass of [M + H]^+^*m/z*^+^ ∼ 271.0608 with λ_max_ ∼ 254 and 336 nm corresponding to molecular formula C_15_H_11_O_5_ for which the calculated mass [M + H]^+^*m/z*^+^ ∼ 271.0606 (Additional file 1: Figure S1), and *t*_*R*_ ~ 15.274 min (P3) with exact mass of [M + H]^+^*m/z*^+^ ∼ 287.0540 with λ_max_ ∼ 247 and 339 nm corresponding to molecular formula C_15_H_11_O_6_ for which the calculated mass [M + H]^+^*m/z*^+^ ∼ 287.0556 (Additional file 1: Figure S1). Peak P2 exactly matched with the *t*_*R*_ of standard apigenin and mass spectra were also similar (Additional file 1: Figure S2). Thus, this product was confirmed to be apigenin while P3 was confirmed to be hydroxylated apigenin, which was predicted to be luteolin. This result showed that M15 could be applied for flavonoids compound not only as monooxygenase but also as flavone synthases [34]. Three recombinant hosts were used for the comparative study of a higher bioconversion of naringenin to the target product with a substrate concentration of 50 µM at 48 h. The comparative conversion rate analysis showed the maximum conversion was 34.48 % by M13, followed by M15 and CYP450 BM3 with 16.11 % and 2.47 %, respectively (Fig. 4a). These results further indicated that M13 harboring strain could be a good recombinant host system for the production of hydroxylated product from naringenin.

### Bioconversion with different naringenin concentration

To study the optimal substrate concentration, we separately supplied the various concentrations of naringenin (50, 100, 200, 300, 500, 800 µM) to biocatalysis reaction system with M13. The cell growth and substrate conversion were monitored at 12 h intervals. The maximum bioconversion of naringenin showed 43.73 % at 48 h with OD_600_ ~ 2.80 when 100 µM of naringenin was added in the biotransformation reaction. Even though the conversion decreased when a higher amount of naringenin was supplied at the same time (Fig. 4b), the growth rate of the cell was steady and stable (Fig. 4c). These results indicated that the high concentration of naringenin could be a reason for inhibition of the hydroxylation product in *E. coli* whole cell.

### Scale-up by fermentation

We cultured the recombinant strain M13 in a 3-L fermentor as described in the materials and methods, where we supplemented an optimized concentration of naringenin (100 µM, ~81.7 mg in 3 L) and glucose 2 %. The temperature of the fermentor was maintained at 28 °C while pH was 7.4 throughout the process. The culture medium was harvested at regular time interval of 12 h and analyzed by HPLC-PDA to monitor the conversion of naringenin to hydroxylated product. The added naringenin was not completely converted until 48 h. Only 46.95 µM (~40.7 mg) of hydroxylated naringenin was produced as a maximal yield of the approximately 49.81 % bioconversion rate of the substrate (Fig. 4d).

### Structural elucidation of hydroxylated naringenin

We analyzed the structures of naringenin standard and purified hydroxylated product by ^1^H-NMR as well as ^13^C-NMR at 700 MHz in DMSO-*d*_*6*_. The ^1^H-NMR spectrum of hydroxylated naringenin showed the absence of proton signal at *δ* = 6.8 ppm (m) for C-3′. Moreover, we observed an upfield shift at *δ* = 146.18 ppm of the C-3′ of hydroxylated product compared to the same carbon of naringenin at *δ* = 115.63 ppm, and an accompanying downfield shift of the resonances of the adjacent carbons C-4′ at *δ* = 145.65 ppm and *δ* = 158.20 ppm, respectively (Table 2; Additional file 1: Figures S8, S9). By these analyses, we confirmed naringenin, which previously has been found in citrus plants [37]. Similarly, we identified 3′,4′,5,7-tetrahydroxyflavanone as a 3′-OH hydroxylated derivative of naringenin, which is known as eriodictyol. The NMR data were consistent with the previously published results [38].

**Table 2:** Comparison of ^1^H-NMR and ^13^C-NMR of naringenin standard with hydroxylated naringenin (eriodictyol)

Carbon no.	Naringenin		Hydroxylated product (Eriodictyol)	
	^1^H NMR	^13^C NMR	^1^H NMR	^13^C NMR
2	5.44 (dd, *J* = 12.8, 3.0 Hz)	78.90	5.38 (dd, *J* = 12.5, 3.1 Hz)	78.92
3-*trans*	3.27 (dd, *J* = 17.1, 12.8 Hz)	42.44	3.19 (dd, *J* = 17.1, 12.5 Hz)	42.54
3-*cis*	2.69 (dd, *J* = 17.1, 3.1 Hz)	42.44	2.68 (dd, *J* = 17.1, 3.2 Hz)	42.54
4		196.88		196.84
4a		102.24		102.26
5-OH	12.16 (s)	163.96	12.15 (s)	163.94
6	5.89 (s)	96.25	5.88 (m)	96.21
7-OH	10.78 (s)	167.11	10.80 (s, 1H)	167.10
8	5.89 (s)	95.43	5.88 (m)	95.41
8a		163.41		163.37
1′		129.32		129.91
2′	7.32 (m)	128.82	6.88 (s)	115.79
3′	6.80 (m)	115.63		146.18
3′-OH			9.05 (d, *J* = 34.2 Hz)	
4′-OH	9.59 (s)	158.20	9.05 (d, *J* = 34.2 Hz)	145.65
5′	6.80 (m)	115.63	6.75 (s)	114.81
6′	7.32 (m)	128.82	6.75 (s)	118.41

*s* singlet, *d* doublet, *dd* doublet of doublet, *m* multiplet

### Antibacterial activities of compounds

We determined the antibacterial activity of flavanone, hesperetin, naringenin, and eriodictyol by disc diffusion assays against five different human pathogens including three Gram-positive (*B. subtilis, M. luteus* and *S. aureus)* and two Gram-negative (*P. aeruginosa* and *E. cloacae*) bacteria in comparison with kanamycin. Additional file 1: Table S1 summarizes the results. Flavanone, hesperetin and naringenin did not display antibacterial activity against the tested Gram-positive bacteria as compared with eriodictyol and kanamycin when 10 µL of 50 mM concentration was applied in disc diffusion. However, the results also showed that flavanone and hesperetin exhibited very low antibacterial activity against Gram-negative bacteria such as *P. aeruginosa* and *E. cloacae*. Interestingly, eriodictyol showed the highest activity in inhibiting *M. luteus*, followed by *S. aureus* and was also equipotent against *B. subtilis,* with zone of inhibition values of 22.5 ± 0.47, 16 ± 0.16 and 11 ± 0.12 mm, respectively. Moreover, we detected the zone of inhibition against *M. luteus* by eriodictyol was 2.5 times higher than kanamycin (Additional file 1: Table S1). The results revealed that the modification of naringenin at the C-3′ position could be beneficial for the enhancement of antibacterial activity against particular Gram-positive bacteria.

### Anticancer activities of compounds

**Fig. 5:**
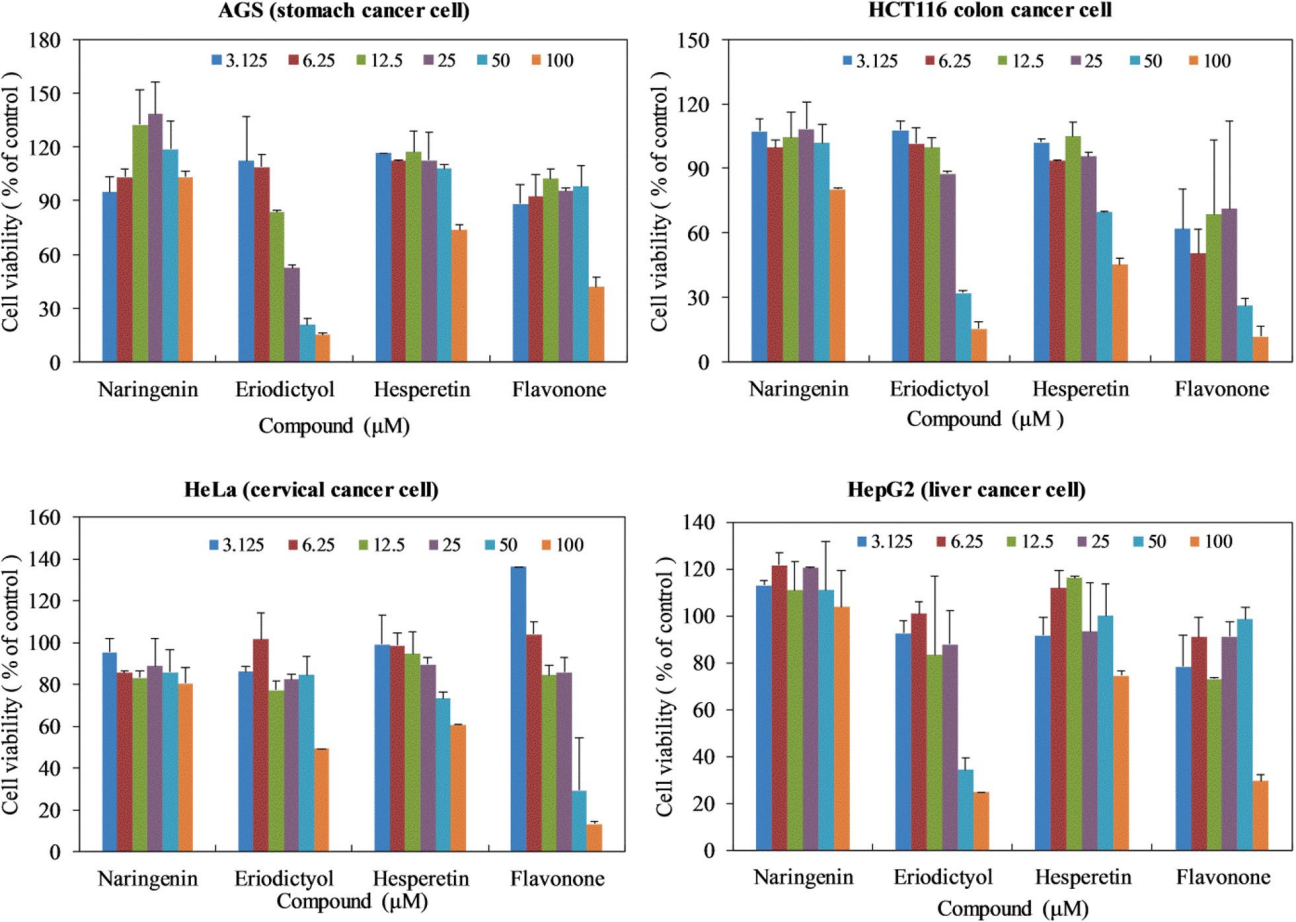
Effects of naringenin, eriodictyol, hesperetin and flavanone on the growth of various cancer cell lines. Cell viability was measured using MTT colorimetric assay. Standard deviations were calculated from the results of three independent experiments

We carried out MTT (3-(4,5-dimethylthiazol-2-yl)-2,5-diphenyltetrazolium bromide) colorimetric assay to evaluate the potential anticancer effects of naringenin, flavanone, hesperetin and eriodictyol on four different cancer cell lines of AGS, HCT116, HepG2, and HeLa. The cell viability data showed that naringenin and hesperetin did not have anticancer activity against all cell lines, whereas eriodictyol exhibited the most effective anticancer activity against four cell lines, which was followed by flavanone (Fig. 5). The cell viability data of AGS, HCT116, Hela, and HepG2 cell lines indicated 100 µM of eriodictyol reduced the cell viability by approximately 15.18 %, 15.28 %, 49.30 %, and 24.94 % (*p* < 0.01), respectively, as compared to control. The 50 % inhibitory concentration (IC_50_) values of eriodictyol were 19.64, 35.85 and 37.72 µM in AGS, HCT116 and HepG2, respectively. In addition, flavanone inhibited HCT116 and Hela cell lines with IC_50_ values of 48.10 and 33.40 µM, respectively (Additional file 1: Table S2). These results suggest that treatment with eriodictyol and flavanone significantly reduces the cell viability of HCT116 and Hela cell lines in a dose-dependent manner. This is the first report of activity of both compounds against HCT116 and Hela cell lines.

### Modeling and docking of naringenin to P450 BM3 and its variants

**Fig. 6:**
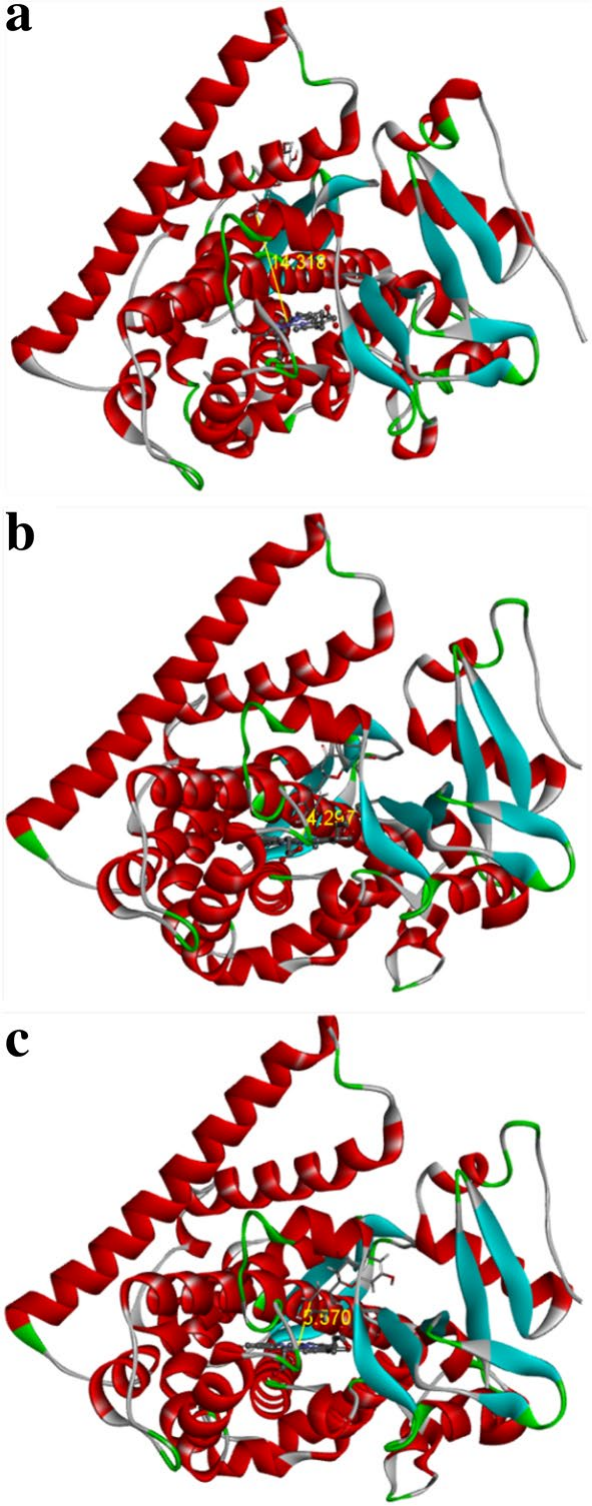
Molecular modeling and docking of naringenin. **a** CYP450 BM3 wild type (Protein Data Bank code: 1BU7), **b** M13 and **c** M15. The average distance from naringenin C-3′ to the Fe of heme in the orientation of 3′-hydroxylation is 14.318, 4.297 and 5.570 Å for CYP BM3, M13 and M15, respectively

We selected a protein crystal structure previously resolved (PDB ID: 1BU7) from *B. megaterium* as template to build the protein models of M13 and M15 using Accelrys Discovery Studio 3.5 software (Accelrys Inc., San Diego, CA). Figure 6 presents three-dimensional (3D) structure of the heme-Fe-dependent monooxygenase domain of each protein. To the models, following active-site optimization, molecular dynamics were used to dock naringenin. The study of all docked conformations of naringenin in the three CYP450 BM3 models showed that the spatial orientation of the active sites of protein M13 is somewhat different from that of protein M15 and CYP450 BM3. The C-3′ position of naringenin was closer to the Fe of heme of the M13 and M15 than to CYP450 BM3 model. The distance between Fe and C-3′ was 4.297, 5.570 and 14.318 Å (Fig. 6) with M13, M15 and CYP450 BM3, respectively. This in silico docking result is consistent with our hydroxylation reaction elucidating the higher catalytic activity of M13 with naringenin and other flavonoids.

## Discussion

The hydroxylation of secondary metabolites is one of the main modifications that have a profound impact on physical as well as biological changes in the molecules [39]. In this study, we studied expanded substrates flexibility of CYP450 BM3 towards flavonoid aglycones. The oxidative hydroxylation of flavonoids was not shown by wild type CYP450 BM3, however, both engineered variants of the same enzyme namely M13 and M15 accepted various flavonoids and isoflavonoids as substrates for hydroxylation which includes flavone (apigenin), flavanones (naringenin, flavanone), flavonol (3-HF), and isoflavonoids (genistein, daidzein, biochanin A) (Table 1). These results suggest that in vitro enzymatic synthesis using CYP450 BM3 variants opened up prospects for the synthesis of hydroxylated flavonoids, which could be applied to other substrates as well, to generate libraries of compounds.

After in vitro reactions, we applied all three enzymes for whole cell catalysis. Interestingly, CYP450 BM3 exhibited activity towards naringenin with very low conversion rate. Two mutant proteins M13 and M15 overexpressing plasmid harboring strain showed higher catalytic efficiency with naringenin than wild type CYP450 BM3 harboring strain (Fig. 3). One possible reason to explain the lower hydroxylation activity of CYP450 BM3 could be the long-distance between possible oxidizable carbons of flavonoids and the heme iron with its ferric resting state in the enzyme [40] as depicted in molecular modelling and docking of naringenin. For example, the average distance C-3′ of naringenin to the Fe of heme of CYP450 BM3 enzyme in the orientation of 3′-hydroxylation is 14.318 Å; whereas, the distances were 4.297 and 5.570 Å with M13 and M15, respectively (Fig. 6). The variation in distance among the three enzymes could be because of the substitution of several key amino acids of CYP450 BM3 wild type while generating M13 and M15. These substituted amino acids were reported to influence either the substrate selectivity or activity or both of CYP450 BM3. For example, the arginine residues at position 47 (ARG47; R47) are believed to be important for entrance of the substrate to the channel, and to control substrate accessibility to the binding pocket [9]. The amino acid leucine at position 86 (LEU86; L86) is positioned near one of the heme propionate groups, and could affect the heme properties leading to increase of enzyme activities [41]. Phenylalanine 87 (PHE87; F87) is another key residue that is highly conserved. Its mutation could affect activity and stereo-selectivity or regio-selectivity of enzyme [42]. Similarly, glutamic acid 267 (GLU267; E267) is called the solvent channel, which prevents bulk amount of solvent from entering the active site [41]. It is also likely that the substituted key amino acid in the wild type protein could cause the change of CO-difference spectra of M13 and M15. As spectrophotometric evidence for the presence of an active form of CYP450, a peak at 450 nm was observed from a CO-induced difference spectrum in all the proteins. However, the CO-difference spectra of M13 and M15 changed by 1923.077, 472.527 nM/L, respectively, in comparison with wild type 747.253 nM/L (Fig. 2).

One emerging application of CYP450s for the pharmaceutical industry is the generation of large amounts of metabolite drug. However, one concern encountered when applying an in vitro enzymatic synthesis approach mediated by CYP450 monooxygenase is the cost of cofactor NAD(P)H. These cofactors are not economically feasible, and unstable in solution [43]. Although the in vitro reaction mixture used a NADPH regenerating system which consists of glucose-6-phosphate dehydrogenase, glucose-6-phosphate, NADP^+^, the cost of the reaction is higher and reaction yield is relatively low. For instance, M13 was found to convert naringenin, flavanone, genistein, daidzein, apigenin, and 3-HF to their respective hydroxylated derivatives with conversion rate of 15.16, 30.78, 36.15, 18.9, 4.89 and 4.47 %, respectively, in the in vitro reaction. While M15 exhibited much lower conversion of those substrates than M13 and CYP450 BM3 did not show any conversion of flavonoids (Table 1). Since, there was very low conversion rate for each substrate; we sought to develop a whole cell approach using the recombinant M13 strain to modify one of the substrates, naringenin for the hydroxylation and production of eriodictyol utilizing endogenous NADPH from *E. coli* cell cytosol. In the whole cell biocatalysis system, production of eriodictyol increased by up to 34.48 % (Fig. 4a). This result indicated that the recombinant M13 strain not only led to the reduction of the cost of reaction but also increased the efficiency of the regio-selective hydroxylation of naringenin. The recombinant M13 strain was further applied for the large-scale production of eriodictyol in a 3-L fermentor supplementing 100 µM of naringenin. The bioconversion experiment produced 46.95 µM (~40.7 mg) of eriodictyol in 48 h incubation (Fig. 4b). These data indicate that the system’s efficiency increased, and could be applicable to high-scale biotransformation. Moreover, metabolic engineering and other approaches are warranted to further enhance the production, and apply to industrial production of the target molecule. For example, overexpression of glucose-6-phosphate dehydrogenase (*zwf*) to redirect the glycolysis flux to the pentose phosphate pathway [44, 45] or the using a stoichiometric-based model to identify combination of gene knockouts in *E. coli*, resulted in significantly improved availability of NADPH in connection to the target production pathway [46].

Another interesting result we observed during this experiment was generation of apigenin in whole cell biotransformation of naringenin while using the recombinant M15 strain. Instead of production of hydroxylated derivative, we were able to detect apigenin and its hydroxylated derivative in the reaction mixture (Fig. 3). Thus, the modification reaction is divided via two paths, the formation of eriodictyol from naringenin which proceeds through hydroxylation directed at C-3′ of B-ring; and M15, which is capable of functioning as flavone synthase on the formation of apigenin from naringenin [34]. However, detailed study on the formation of apigenin and its hydroxylated derivative from naringenin are essential and which we will be carried out in the near future using M15.

We also accessed the antibacterial and anticancer activities of three flavanones and eriodictyol in this study. Three Gram-positive bacteria *M. luteus, B. subtilis,* and *S. aureus* were sensitive with eriodictyol, while two Gram-negative bacteria *P. aeruginosa* and *E. cloacae* were susceptible with flavanone and hesperetin. Interestingly, eriodictyol showed higher antibacterial activity against *M. luteus* than an aminoglycoside antibiotic, kanamycin (Additional file 1: Table S1). The possible reason behind the antibacterial activity of eriodictyol could be the intercalation or formation of hydrogen bond between B ring of the eriodictyol and stacking between nucleic acid bases, which lead to the inhibition of DNA and RNA synthesis in bacteria [47]. Among four compounds, eriodictyol and flavanone showed the most potential anticancer activity against cell lines (Fig. 5; Additional file 1: Table S2). Previous study reported that eriodictyol inhibited the production of TNF-α and IL-β in kidney tissues, and inhibited the production of blood urea nitrogen (BUN), as well as up-regulating the expression of nuclear factor erythroid 2-related factor 2/Heme oxygenase-1 and inhibiting of cisplatin-induced nuclear factor-kappa B activation in kidney tissues [48]. In addition to those activities, we accessed the activity of eriodictyol against HCT116 and Hela cell lines for the first time. These results collectively open up the possible benefits of hydroxylation of naringenin at the C-3′ position to generate promising lead molecule against various cancer cell lines as well as Gram-positive bacteria.

## Conclusions

In conclusion, the present study shows that protein engineering of CYP450 BM3 enzyme is able to expand the substrate flexibility of enzyme towards the flavonoid group of compounds. Moreover, the study also demonstrated the application of engineered protein in whole cell biocatalysis for the production of valuable molecules by a simple fermentation approach, which is very easy to scale up, and cheaper than other approaches. As a proof of study, we used naringenin as a substrate for hydroxylation in in vivo system and demonstrated the production of eriodictyol in a lab scale fermentor. Though the production of the target hydroxylated molecule was low, the application of metabolic engineering and synthetic biology tools will certainly help to enhance the production of eriodictyol from naringenin. This is the first report of eriodictyol being produced from naringenin using recombinant CYP450 BM3 and its variants from *B. megaterium*. We believe that this study opens up an approach for the production of important hydroxylated compounds of various polyphenols in an industrial scale using CYP450 BM3 variant which is restricted to fatty acids.

## Methods

### Chemicals and reagents

β-Nicotinamide adenine dinucleotide phosphate sodium salt hydrate in oxidized form was bought for biochemical research from Tokyo Chemical Industry Co., Ltd (Kita-Ku, Tokyo, Japan). HPLC grade acetonitrile and water were purchased from Mallinckrodt Baker (Phillipsburg, NJ, USA). All the flavonoids and chemicals used were purchased from Sigma–Aldrich Chemical Co. (St. Louis, MO, USA).

### Plasmids, microorganisms and media

The plasmid pCW(Ori^+^)-CYP450 BM3 wild type, pCW(Ori^+^)-mutant 13 (M13) and pCW(Ori^+^)-mutant 15 (M15) have previously been constructed [13]. Each mutant carries the following amino acid substitution relative to wild type CYP450 BM3: M13 (R47L/L86I/F87V/L188Q) [49] and M15 (R47L/E64G/F87V/E143G/L188Q/E267V) [13]. We transformed these plasmids into *E. coli* BL21 (DE3) (Stratagene, USA) using standard procedures. The 5× M9 salts stock solution contained 30 g Na_2_HPO_4_, 15 g KH_2_PO_4_, 2.5 g NaCl, 5 g NH_4_Cl, 0.24 g MgSO_4_, 0.01 g CaCl_2_ and 20 g glucose was used to prepare 1× M9 minimal medium for bacterial culture when needed.

### Protein expression and purification of P450 BM3

We cultured the recombinant strains in 5 mL Luria–Bertani (LB) medium supplemented with ampicillin (100 μg/mL) at 37 °C, 180 rpm for 6 h. For protein overexpression, the pre-inoculum (200 µL) was transferred into a 250 mL shaking flask containing 50 mL 1× M9 minimal medium supplemented with ampicillin and incubated at 37 °C, 180 rpm. When the optical density at 600 nm (OD_600_ nm) reached 0.8, the protein expression was induced with 0.5 mM final concentration of isopropyl-β-d-thiogalactopyranoside (IPTG). To the culture, 1 mM δ-aminolevulinic acid hydrochloride (δ-ALA) was added and incubated for 36 h at 28 °C. The cells were harvested by centrifugation at 842×*g* for 15 min, and washed twice with 50 mM phosphate buffer pH 7.4 containing 10 % glycerol (PG buffer) for two times. The cells were suspended in 1 mL of the same buffer containing 1 mM dithiothreitol (DTT) and 1 mM phenylmethylsulfonyl fluoride (PMSF). The cells were lyzed by sonication using a Sonosmasher (2 × 1 min, output control 5, 50 % duty cycle; Sonicator, Heat Systems, Ultrasonic, Inc.). Following centrifugation at 13,475×*g* for 30 min at 4 °C, the resulting soluble and insoluble protein fractions were analyzed by 12 % sodium dodecyl sulfate polyacrylamide gel electrophoresis (SDS–PAGE). We collected the soluble cytosolic fraction using Amicon^®^Ultra 50 mL filters (Millipore, 100,000 K NMWL device; Milford, MA, USA) and stored it in PG buffer at −20 °C until the activity assay.

### Determination of protein concentration

We determined CYP450 BM3 protein and variants (M13 and M15) content as described by Omura and Sato [50] using a Shimadzu 1601PCs spectrophotometer (Tokyo, Japan). The content of protein CYP450 BM3 was calculated from the spectral difference of extinction CO-efficient at 450 and 490 nm of € = 91 mM^−1^cm^−1^. The protein CYP450 BM3 content (nmol) = [(absorbance difference × 1000)/91] dilution factor. We determined the protein concentration by Bradford method using bovine serum albumin as the reference standard [51].

### In vitro reaction condition

We carried out 250 µL reactions using the protein CYP450 BM3, M13, M15 and 0.5 mM substrate in potassium phosphate buffer (100 mM, pH 7.4) and 10 mM MgCl_2_·6H_2_O. The reaction was incubated for 15 min at 37 °C with individual substrates naringenin, flavanone, flavone, chrysin, luteolin, diosmetin, baicalein, 7,8-dihydroxy flavone, morin, genistein, daidzein, biochanin A, kaempferol, quercetin, fisetin, and myricetin in order to investigate the substrate specificity of the enzyme (Fig. 1). A NADPH regenerating system consisting of glucose-6-phosphate-dehydrogenase (0.5 U), glucose-6-phosphate (10 mM), and NADP^+^ (0.5 mM) was then added to the reaction mixture and incubated for 30 min at 37 °C with shaking. The reaction was stopped by adding a double volume of chilled ethyl acetate, followed by vigorous shaking for 30 min, and centrifuged at 13,475×*g* for 10 min. The ethyl acetate organic layer was collected and concentrated to dryness by the evaporation of excess solvent. The samples were dissolved in 250 µL methanol, and analyzed by high performance liquid chromatography-photodiode array (HPLC-PDA) and high-resolution quadruple time-of-flight electrospray ionization-mass spectrometry (HR-QTOF ESI/MS). The conversion percentage of each substrate was determined after integrating product and substrate peaks. For quantification of flavonoids, we created a calibration curve of authentic substrate using 10, 25, 50, 100 and 200 μg/mL concentrations.

### Kinetics measurement for naringenin oxidation reactions

We determined the kinetic parameters of M13 and M15 proteins by using reaction mixtures containing variable amounts of naringenin (from 0.05 to 2 mM) in potassium phosphate buffer (100 mM, pH 7.4) consisting of 10 mM MgCl_2_·6H_2_O at 37 °C. We analyzed the products by HPLC-PDA and the *K*_*m*_ and *V*_*max*_ values by the Lineweaver–Burk plotting method.

### Whole cell biotransformation of naringenin

We precultured the CYP450 BM3, M13 and M15 strains in 5 mL LB liquid medium with ampicillin, and incubated them at 37 **°**C with shaking at 180 rpm for about 6 h. For whole cell reaction, 200 µL of pre-inoculum was transferred into 250 mL flask containing 50 mL of 1× M9 minimal medium with appropriate antibiotic. The cells were incubated in shaking incubator at 37 °C. When the OD_600_ reached 0.8, the culture was induced with 0.5 mM final concentration of IPTG. 1 mM δ-ALA was also added and incubated for 12 h at 28 °C. To the same culture, standard naringenin dissolved in 10 % dimethyl sulfoxide (DMSO) was supplemented at a final concentration of 50 µM and extended to incubate at 28 °C for 60 h. Every 12 h, we collected 500 µL of culture, extracted and analyzed by HPLC-PDA.

### Scale-up of whole-cell biocatalyst system in a fermentor

For large scale biotransformation and production of hydroxylated naringenin, we carried out fermentation in 3 L of 1× M9 minimal media under previously reported conditions [52] using recombinant strain harboring M13 gene overexpressing plasmid. To this induced culture 100 µM of naringenin was added for biotransformation. The culture was supplied with 20 mL sterile glucose solution (600 g/L) for every hour until 36 h after the start of feeding of naringenin. We took samples at every 12 h intervals until 48 h for the quantification of product formation.

### Analytical methods

We obtained the crude extract of compound after extracting culture broth with double volume of ethyl acetate (v/v = 2:1) in Soxhlet extractor. The mixture was shook for 12 h at room temperature. Then, Soxhlet extractor was kept still, and stood until separated into two layers. Ethyl acetate organic fraction was taken and dried by freezing rotary evaporator. The product formation was analyzed by HPLC-PDA using a reversed-phase column (Mightysil RP–18 GP 250–4.6 (5 µm), Kanto Chemical, Japan) at UV absorbance of 290 nm. The binary mobile phases were composed of solvent A (0.05 % trifluroacetic acid (TFA) in HPLC-grade water) and solvent B (100 % acetonitrile, CH_3_CN). The total flow rate was maintained at 1 mL/min for a 30 min. The percentage of solvent B used was as follows: 0–20 % (0–5 min), 50 % (5–10 min), 70 % (10–15 min), 90 % (15–20 min), 10 % (20–25 min), 10 % (25–30 min). The purification of compounds was carried out by preparative HPLC (Shimadzu, Tokyo, Japan) with C_18_ column (YMC–Pack ODS-AQ (150 × 20 mm I.D., 10 µm) connected to a UV detector at a UV absorbance of 290 nm using a 36-min binary program with CH_3_CN 20 % (0–5 min), 40 % (5–10 min), 40 % (10–15 min), 90 % (15–25 min), 90 % (25–30 min) and 10 % (30–36 min) at a flow rate of 10 mL/min. The HR-QTOF ESI/MS analysis was performed in positive ion mode using an ACQUITY (UPLC, Waters Corp., Billerica, MA, USA) column coupled with a SYNAPT G2-S (Water Corp.). For nuclear magnetic resonance (NMR) analysis of the purified product, compounds were dried, lyophilized, and dissolved in dimethyl sulfoxide (DMSO)-*d*_*6*_ and subjected to 700 MHz Bruker, Biospin NMR for one-dimensional ^1^H-NMR, ^13^C-NMR.

### Biological activities

#### Antibacterial activity

To evaluate the biomedical application of flavanone, hesperetin, naringenin and eriodictyol as a antibacterial agent, we employed the paper disc diffusion method on Mueller–Hinton agar (MHA) plates using kanamycin as antibacterial agent control. Three Gram-positive bacteria *Staphylococcus aureus* subsp. *aureus* KCTC 1916, *Bacillus subtilis* KACC 17047, *Micrococcus luteus* KACC 13377, and two Gram-negative bacteria *Pseudomonas aeruginosa* KACC 10232 and *Enterobacter cloacae* subsp. *dissolvens* KACC 13002 were used. The inocula containing 10^7^ colony forming units (CFU)/mL were spread on MHA plates for bio-assay. Sterile filter paper discs (6 mm in diameter) containing 10 µL of 50 mM compounds were placed on the surface of the inoculated agar plates. The plates were incubated at 37 °C for 12 h. We tested each compound in triplicate, and measured the zone of inhibition in millimeter diameter [53].

#### Anticancer activities

To evaluate the pharmaceutical potential of naringenin, eriodictyol, flavanone and hesperetin as a chemotherapeutic agent for cancer treatment, we used various cancer cell lines consisting of AGS, HCT116, HepG2 and HeLa in Roswell Park Memorial Institute 1640 medium (Invitrogen, Grand Island, NY, USA) containing 10 % fetal bovine serum (FBS, Invitrogen). All cells were maintained at 37 °C in a humidified 5 % CO_2_ incubator. For cell growth assay, cells seeded at 2 × 10^3^ cell/well in 96-well plates (SPL Life Sciences, Gyeonggi, Korea) were treated with each compound in serial dilution (100, 50, 25, 12.5, 6.25, 3.125 μM) for 72 h. We measured cell growth using a MTT colorimetric assay [54].

### Molecular modeling and docking of naringenin in P450 BM3 and its mutants

We selected CYP450 BM3 protein template (Protein Data bank [PDB] ID: 1BU7; crystal structure resolved at 1.65 Å) from *B. megaterium* to build the model with protein M13 and M15 using Accelrys Discovery Studio v.3.5 (2013) (DS 3.5) (Accelrys, San Diego, CA, USA). The construction and validation of protein M13 and M15 models were carried out as previously described [55]. Naringenin was docked to wild type protein, and the validated models of protein M13 or M15 using the “Ligand-Fit” procedure implemented in DS 3.5. The conformations of each ligand were fitted in the binding cavity of protein with a grid-based energy function [56]. We finally selected the most suitable docking mode for naringenin with a high score from the consensus scoring function to find the distance between heme core and naringenin.

## Additional file

10.1186/s12934-016-0533-4 Inhibition zone diameter (mm) of four flavonoids along with kanamycin against various Gram-positive and Gram-negative bacteria in disc-diffusion assay. **Table S2.** IC_50_ values of four compounds against AGS, HTC116, Hela, HepG2. **Figure S1.** The UV maxima absorbance and exact mass analysis of naringenin (A) and reaction products P1 (B), P2 (C), P3 (D). P1 have been identified as hydroxylated naringenin while P2 and P3 have been identified as apigenin and hydroxylated apigenin, respectively. **Figure S2.** HPLC-PDA, the UV maxima absorbance and HR-QTOF ESI/MS analysis of reaction products and apigenin standard. **Figure S3.** HPLC-PDA, the UV maxima absorbance and HR-QTOF ESI/MS analysis of reaction products and 3-HF standard. **Figure S4.** HPLC-PDA, the UV maxima absorbance and HR-QTOF ESI/MS analysis of reaction products and flavanone standard. **Figure S5.** HPLC-PDA, the UV maxima absorbance and HR-QTOF ESI/MS analysis of reaction products and genistein standard. **Figure S6.** HPLC-PDA, the UV maxima absorbance and HR-QTOF ESI/MS analysis of reaction products and daizein standard. **Figure S7.** HPLC-PDA, the UV maxima absorbance and HR-QTOF ESI/MS analysis of reaction products and biochanin A standard. **Figure S8.** 1-Dimensional NMR of naringenin standard. **Figure S9.** 1-Dimensional NMR of eriodictyol.

## Abbreviations

CYP450 BM3: cytochrome P450 BM3
IPTG: isopropyl-β-d-thiogalactopyranoside
δ-ALA: δ-aminolevulinic acid hydrochloride
HPLC-PDA: high performance liquid chromatography-photodiode array
HR-QTOF ESI/MS: high resolution-quadruple time of flight electrospray ionization/mass spectrometry
DMSO: dimethyl sulfoxide
SDS-PAGE: sodium dodecyl sulfate polyacrylamide gel electrophoresis
AGS: gastric carcinoma
HCT116: colon carcinoma
HepG2: hepatic carcinoma
HeLa: epithelioid cervix carcinoma
