# Supplementary material for: Hydroxylation of diverse flavonoids by CYP450 BM3 variants: biosynthesis of eriodictyol from naringenin in whole cells and its biological activities
Source: Microb Cell Fact. 2016 Aug 5;15:135. doi: 10.1186/s12934-016-0533-4 (PMC4974697; doi:10.1186/s12934-016-0533-4)

**Additional files for:**

*Microbial Cell Factories*

**Hydroxylation of diverse flavonoids by CYP450 BM3 variants: biosynthesis of eriodictyol from naringenin in whole cells and its biological activities**

Luan Luong Chu

[chuluongluan218@gmail.com](mailto:chuluongluan218@gmail.com)

Ramesh Prasad Pandey

[pandey@sunmoon.ac.kr](mailto:pandey@sunmoon.ac.kr)

Narae Jung,

jnr583@naver.com

Hye Jin Jung,

[poka96@sunmoon.ac.kr](mailto:poka96@sunmoon.ac.kr)

Eun-Hee Kim

[keh@kbsi.re.kr](mailto:keh@kbsi.re.kr)

Jae Kyung Sohng*

[sohng@sunmoon.ac.kr](mailto:sohng@sunmoon.ac.kr)

***Corresponding author**: *Department of BT-Convergent Pharmaceutical Engineering,* *Sun Moon University, 70 Sunmoon-ro 221, Tangjeong-myeon, Asan-si, Chungnam 336-708, Republic of Korea.*

Tel: +82(41)530-2246 Fax: +82(41)544-2919

**Abstract**

**Background**: Cytochrome P450 monooxygenase constitutes a significant group of oxidative enzymes that can introduce an oxygen atom in a high regio- and stereo-selectivity mode. We used the *Bacillus megaterium* cytochrome P450 BM3 (CYP450 BM3) and its variants namely mutant 13 (M13) and mutant 15 (M15) for the hydroxylation of diverse class of flavonoids.

**Results**: Among twenty flavonoids, maximum seven flavonoids were hydroxylated by the variants while none of these molecules were accepted by CYP450 BM3 in *in vitro* reaction. Moreover, M13 exhibited higher conversion of substrates than M15 and CYP450 BM3 enzymes. We found that M13 carried out regiospecific 3ʹ-hydroxylation reaction of naringenin with the highest conversion among all the tested flavonoids. The apparent *K_m_* and *k_cat_* values of M13 for naringenin were 446 µM and 1.955 s^-1^, respectively. In whole-cell biotransformation experiment with 100 µM of naringenin in M9 minimal medium with 2% glucose in shake flask culture, M13 showed 2.14 and 13.96 folds higher conversion yield in comparison with M15 (16.11%) and wild type (2.47%). The yield of eriodictyol was 46.95 µM (~ 40.7 mg (13.5 mg/L)) in a 3-L volume lab scale fermentor at 48 h in the same medium exhibiting approximately 49.81% conversion of the substrate. In addition, eriodictyol exhibited higher antibacterial and anticancer potential than naringenin, flavanone and hesperetin against various Gram positive and Gram negative bacteria.

**Conclusions**: We elucidated that eriodictyol being produced from naringenin using recombinant CYP450 BM3 and its variants from *B. megaterium*, which shows an approach for the production of important hydroxylated compounds of various polyphenols in an industrial that may span pharmaceutical industries.

**Keywords:** Cytochrome P450 BM3, monooxygenase, hydroxylation, flavonoids

**Table of contents**

**Table S1.** Inhibition zone diameter (mm) of four flavonoids along with kanamycin against various Gram-positive and Gram-negative bacteria in disc-diffusion assay.

**Table S2.** IC_50_ values of four compounds against AGS, HTC116, Hela, HepG2.

**Figure S1.** The UV maxima absorbance and exact mass analysis of naringenin (**A**) and reaction products P1 (**B**), P2 (**C**), P3 (**D**). P1 have been identified as hydroxylated naringenin while P2 and P3 have been identified as apigenin and hydroxylated apigenin, respectively.

**Figure S2.** HPLC-PDA, the UV maxima absorbance and HR-QTOF ESI/MS analysis of reaction products and apigenin standard. (**A**) HPLC-PDA chromatogram; (**B**) The maxima UV absorbance and exact mass of apigenin and (**C**) hydroxylated apigenin (a1) are shown.

**Figure S3.** HPLC-PDA, the UV maxima absorbance and HR-QTOF ESI/MS analysis of reaction products and 3-HF standard. **(A**) HPLC-PDA chromatogram; (**B**) The maxima UV absorbance and exact mass of 3-HF and (**C**) hydroxylated 3-HF (h1) are shown.

**Figure S4.** HPLC-PDA, the UV maxima absorbance and HR-QTOF ESI/MS analysis of reaction products and flavanone standard. **(A**) HPLC-PDA chromatogram; (**B)** The UV maxima absorbance and exact mass of flavanone, (**C**) mono-hydroxylated (f2), (**D**) mono-hydroxylated (f3) and (**E**) di-hydoxylated (f1) are shown.

**Figure S5.** HPLC-PDA, the UV maxima absorbance and HR-QTOF ESI/MS analysis of reaction products and genistein standard. **(A**) HPLC-PDA chromatogram; (**B**) The UV maxima absorbance and exact mass of standard, (**C**) mono-hydroxylated (g1) and mono-hydroxylated (g2) are shown.

**Figure S6.** HPLC-PDA, the UV maxima absorbance and HR-QTOF ESI/MS analysis of reaction products and daizein standard. **(A**) HPLC-PDA chromatogram; (**B**) The UV maxima absorbance and exact mass of standard, (**C**) and (**D**) two mono-hydroxylated (d1, d2) are shown.

**Figure S7.** HPLC-PDA, the UV maxima absorbance and HR-QTOF ESI/MS analysis of reaction products and biochanin A standard. **(A**) HPLC-PDA chromatogram; (**B**) The maxima UV absorbance and exact mass of standard, (**C**) mono-hydroxylated (b3), (**D**) demethylated (b2) and (**E**) combination between demethylated and hydroxylated (b1) are shown.

**Figure S8.** 1-Dimensional NMR of naringenin standard. (**A**) ^1^H-NMR, (**B**) ^13^C-NMR.

**Figure S9.** 1-Dimensional NMR of eriodictyol. (**A**) ^1^H-NMR, (**B**) ^13^C-NMR.

**Table S1.**

| **Pathogens** | *B. subtilis* | *M. luteus* | *S. aureus* | *P. aeruginosa* | *E. cloaceae* |
| --- | --- | --- | --- | --- | --- |
| Flavanone | **–** | **–** | **–** | **+** | **–** |
| Hesperetin | **–** | **–** | **–** | **+** | **+** |
| Naringenin | **–** | **–** | **–** | **–** | **–** |
| Eriodictyol | 11 ± 0.12 | 22.5 ± 0.47 | 16 ± 0.19 | **–** | **–** |
| Kanamycin | 20 ± 0.27 | 9 ± 0.18 | 19 ± 0.43 | 16 ± 0.36 | 21.5 ± 0.24 |
| *(*–*)* denotes no inhibition zone; (+), inhibition zone detected . Values are mean ± standard deviation of at least three independent experiments (p < 0.05) | | | | | |

**Table S2.**

| **IC_50_ (μM)** | **Naringenin** | **Eriodictyol** | **Hesperetin** | **Flavonone** |
| --- | --- | --- | --- | --- |
| **AGS** | > 100 | 19.64 | > 100 | 98.24 |
| **HCT116** | > 100 | 35.85 | 48.73 | 48.10 |
| **HeLa** | > 100 | 100 | > 100 | 33.40 |
| **HepG2** | > 100 | 37.72 | > 100 | 102.4 |

**Figure S1.**

**
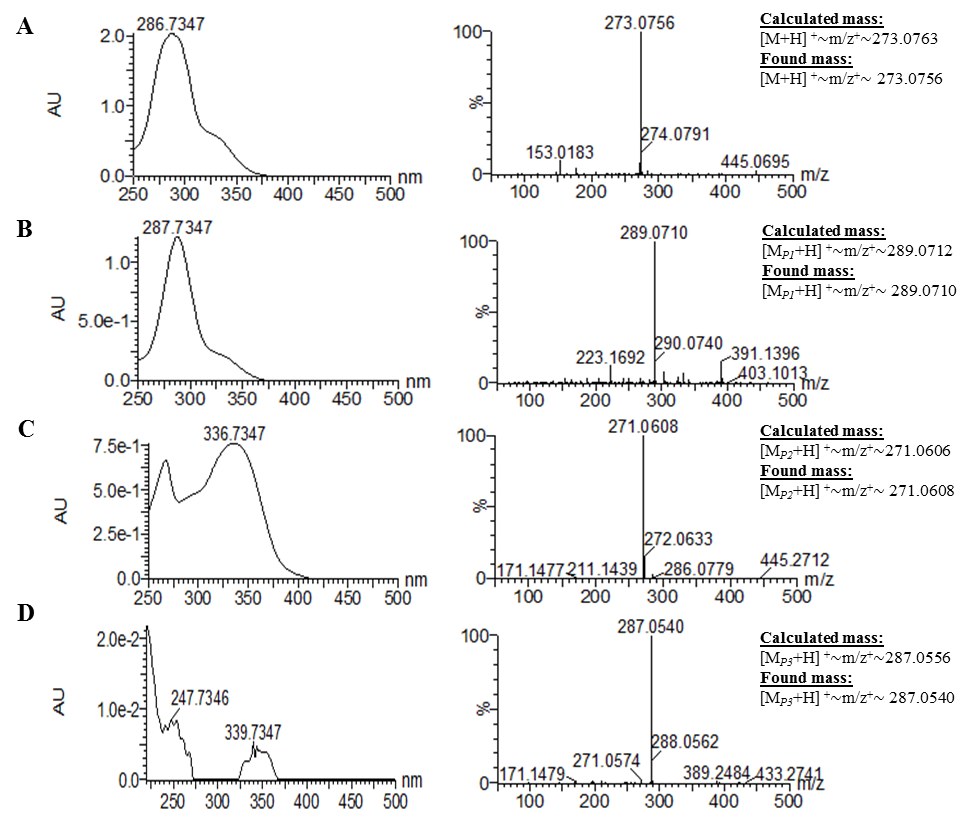
**

**Figure S2**

**
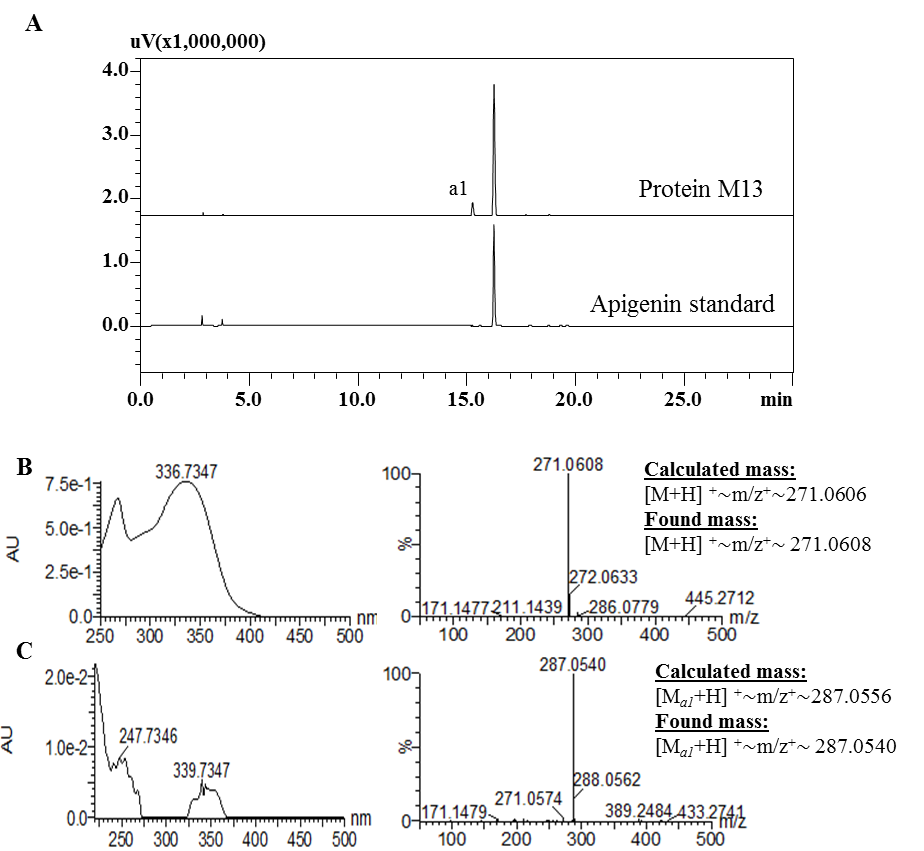
**

**Figure S3**

**
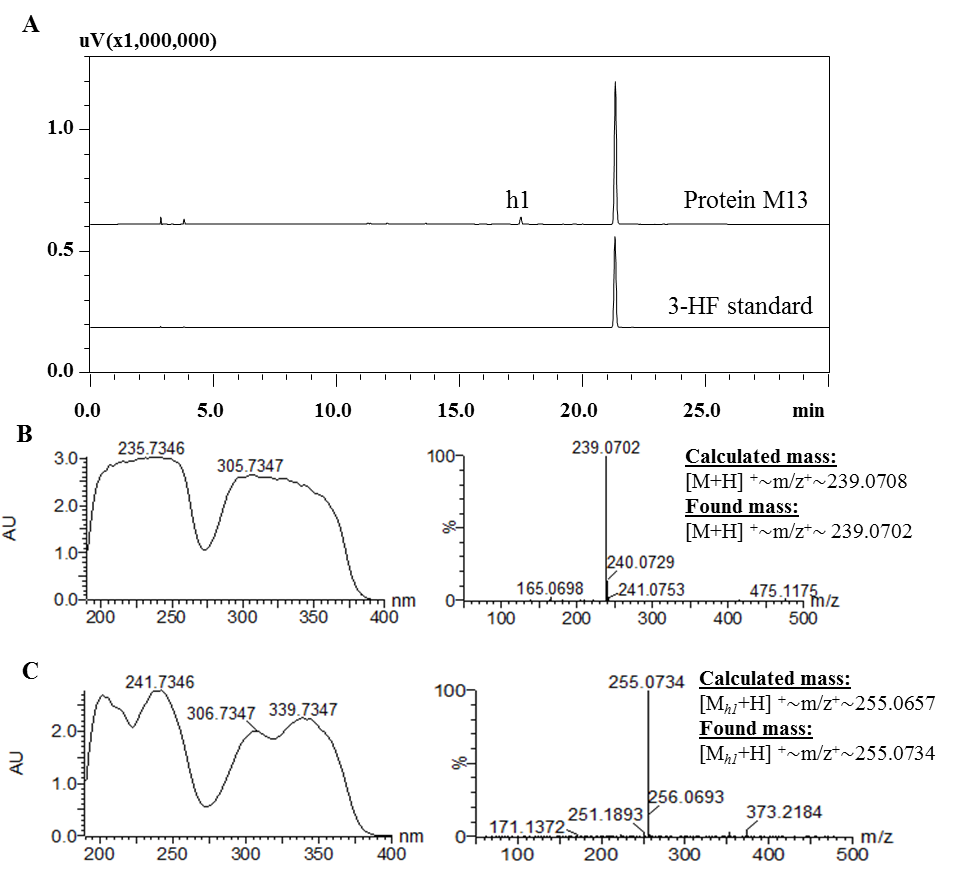
**

**Figure S4**

**
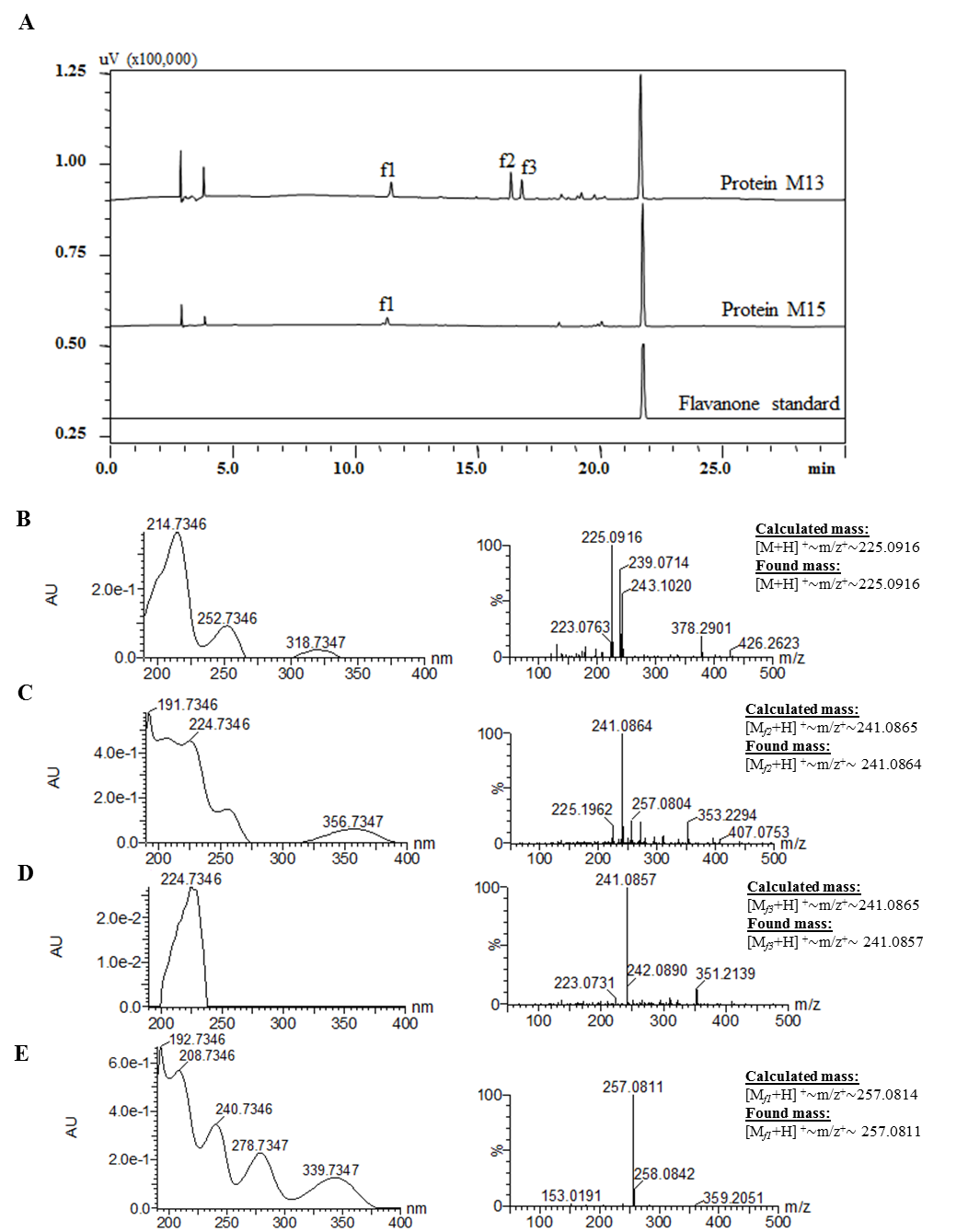
**

**Figure S5**

**
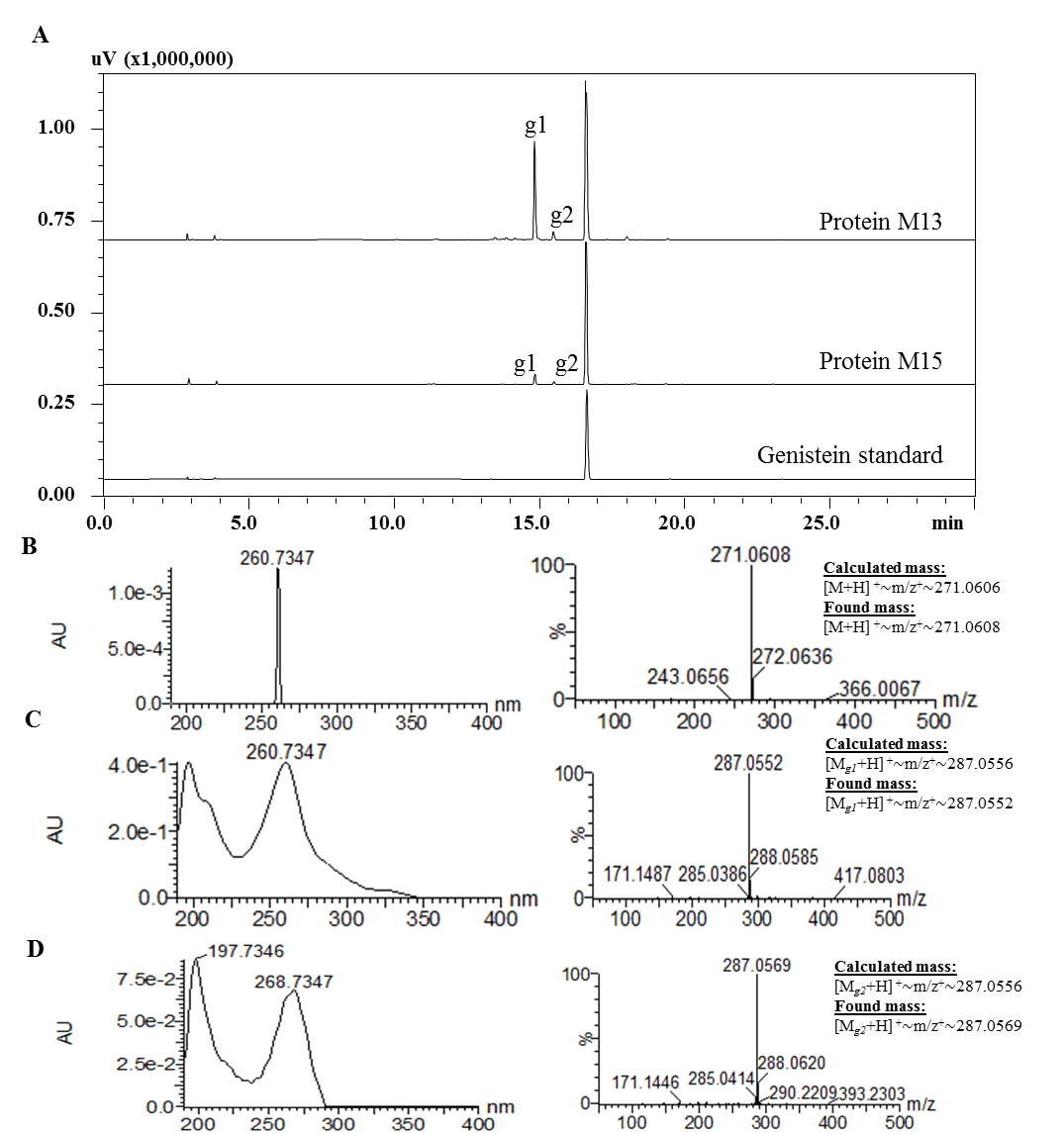
**

**Figure S6**

**
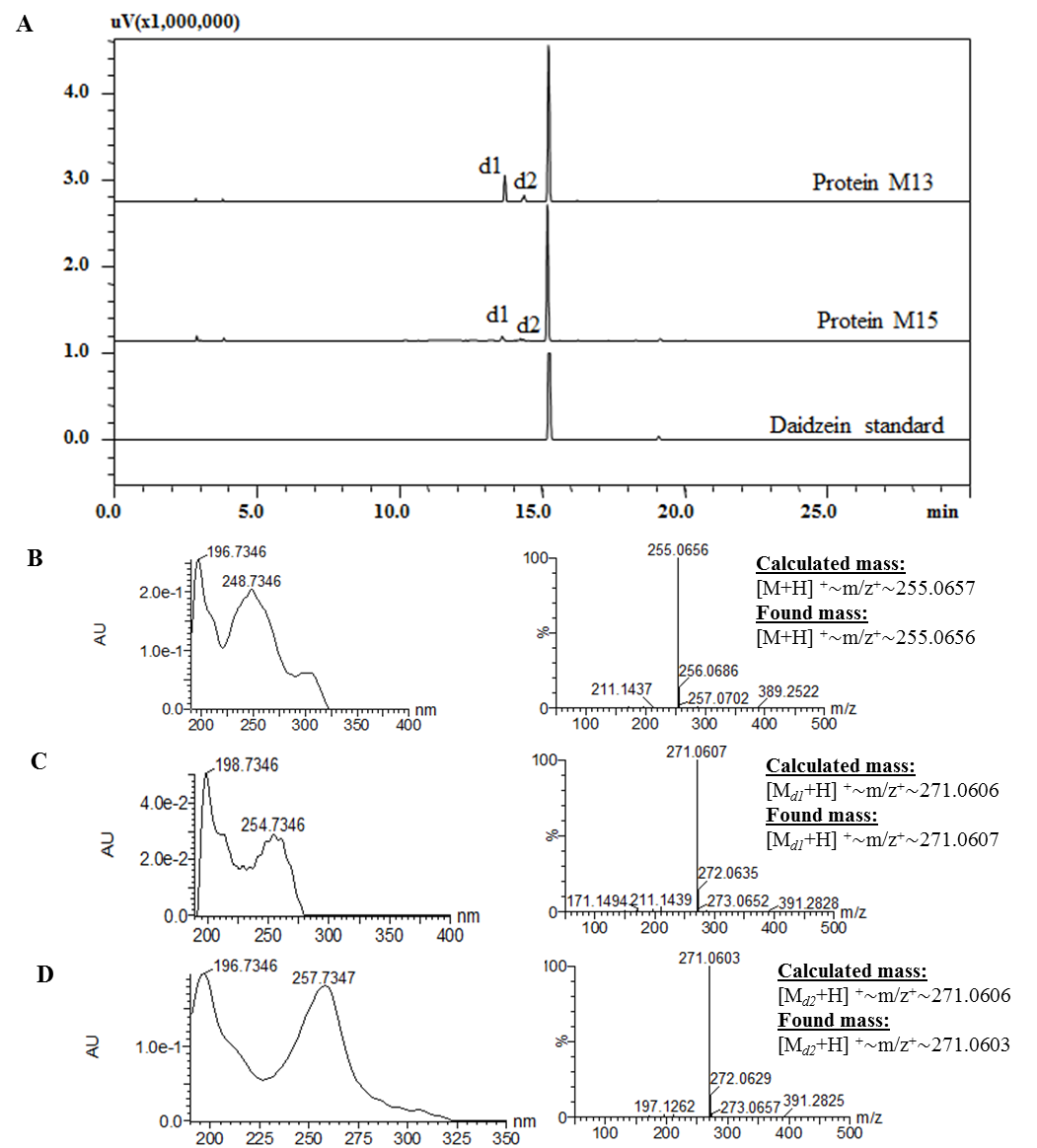
**

**Figure S7**

**
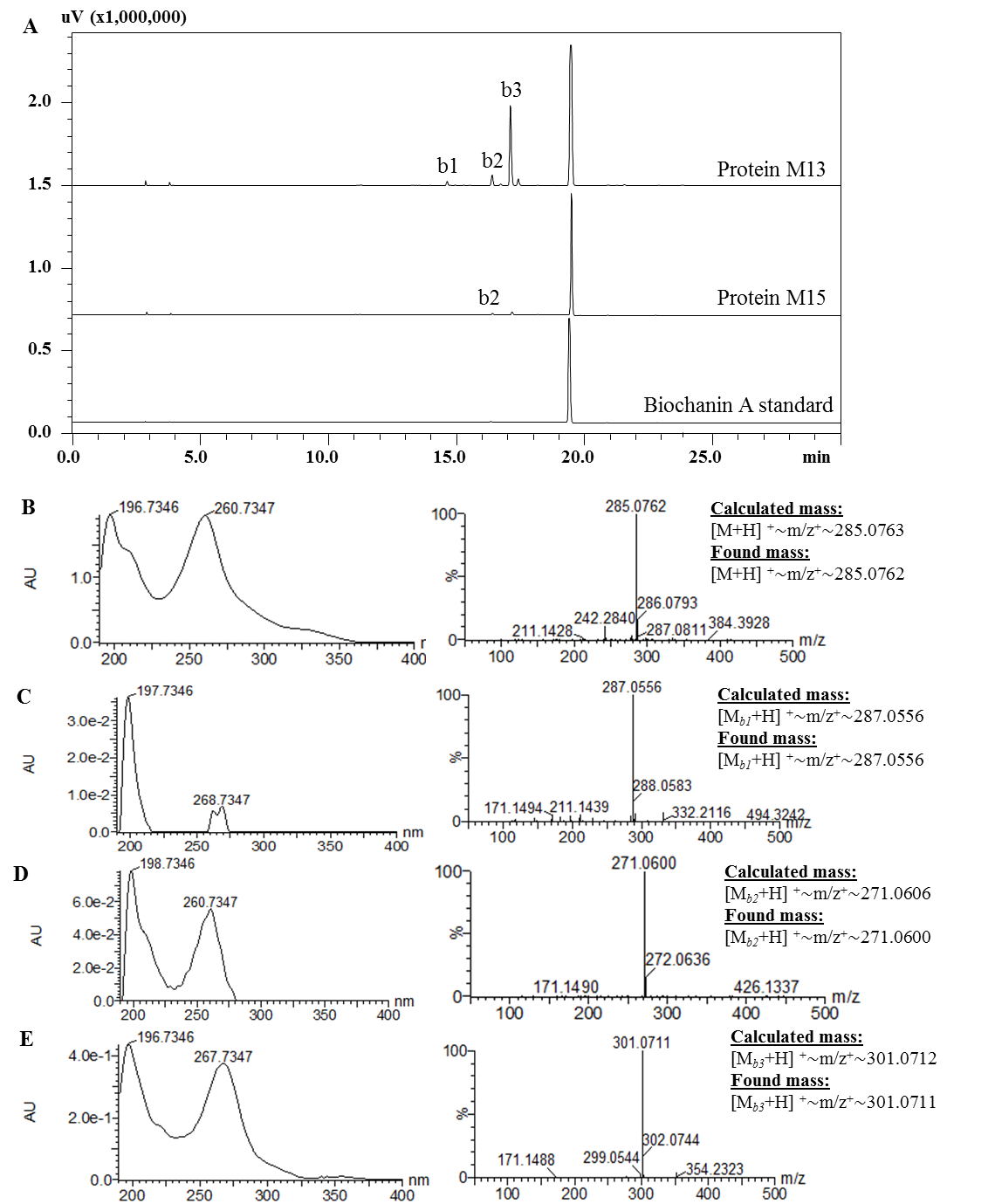
**

**Figure S8.** 1-Dimensional NMR of naringenin standard

A. ^1^H NMR


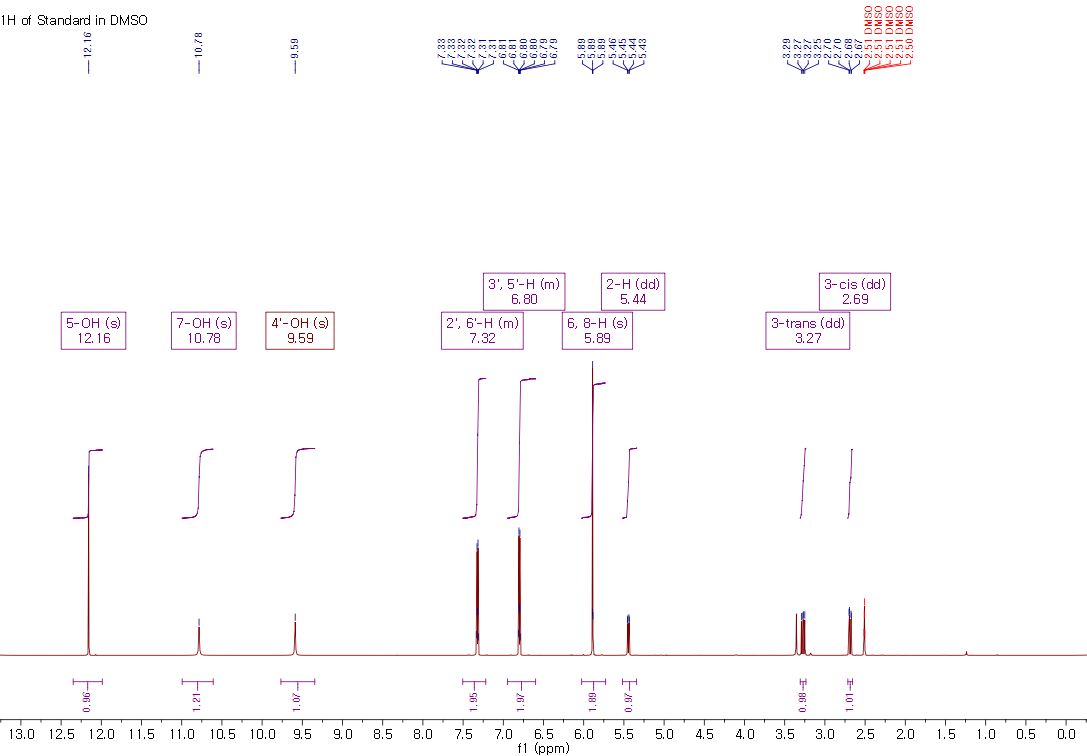


B. ^13^C NMR


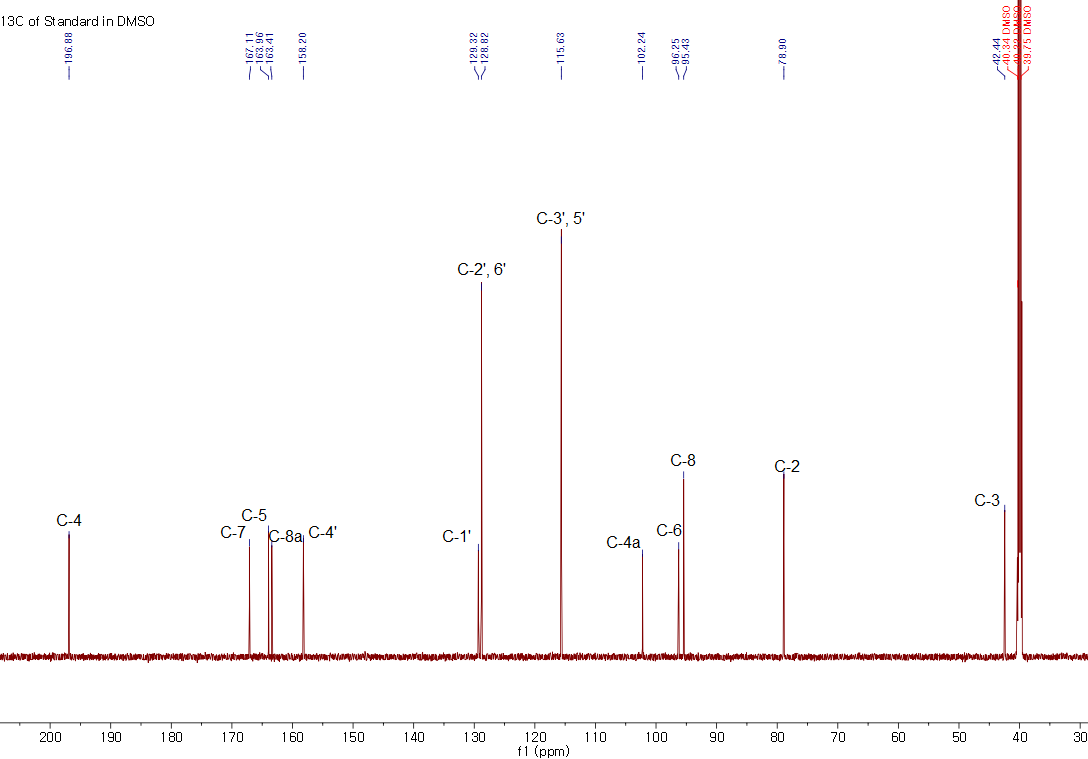


**Figure S9**. 1-Dimensional NMR of eriodictyol

A. ^1^H NMR


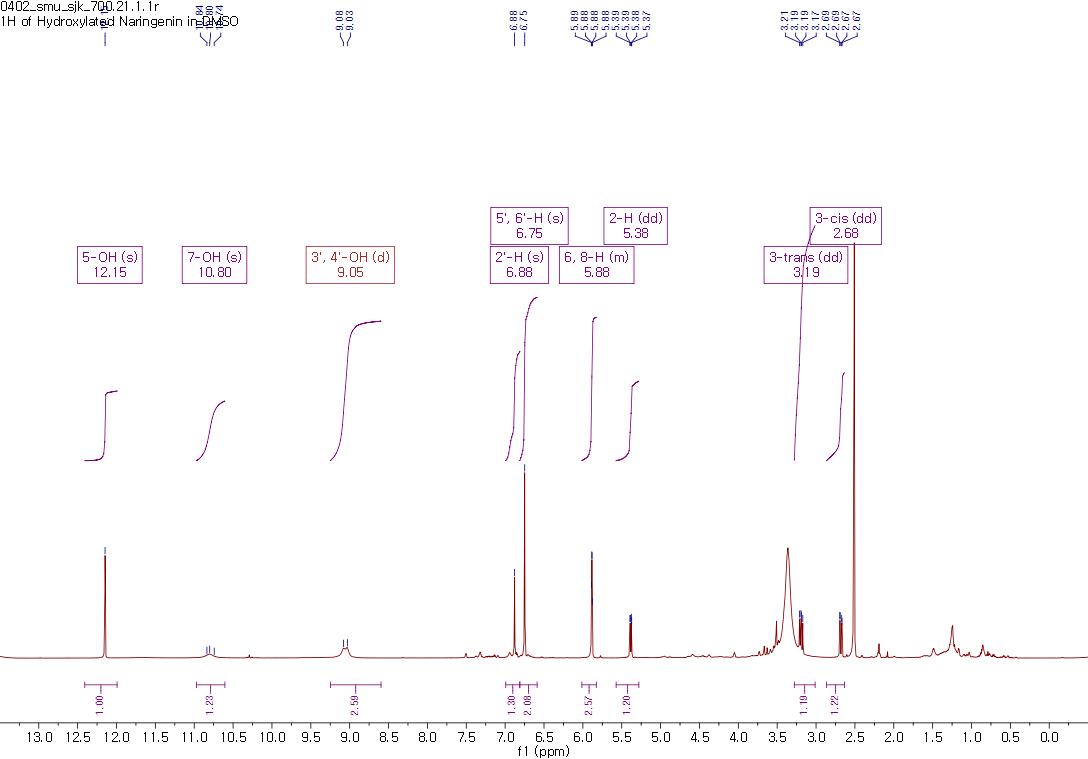


B. ^13^C NMR


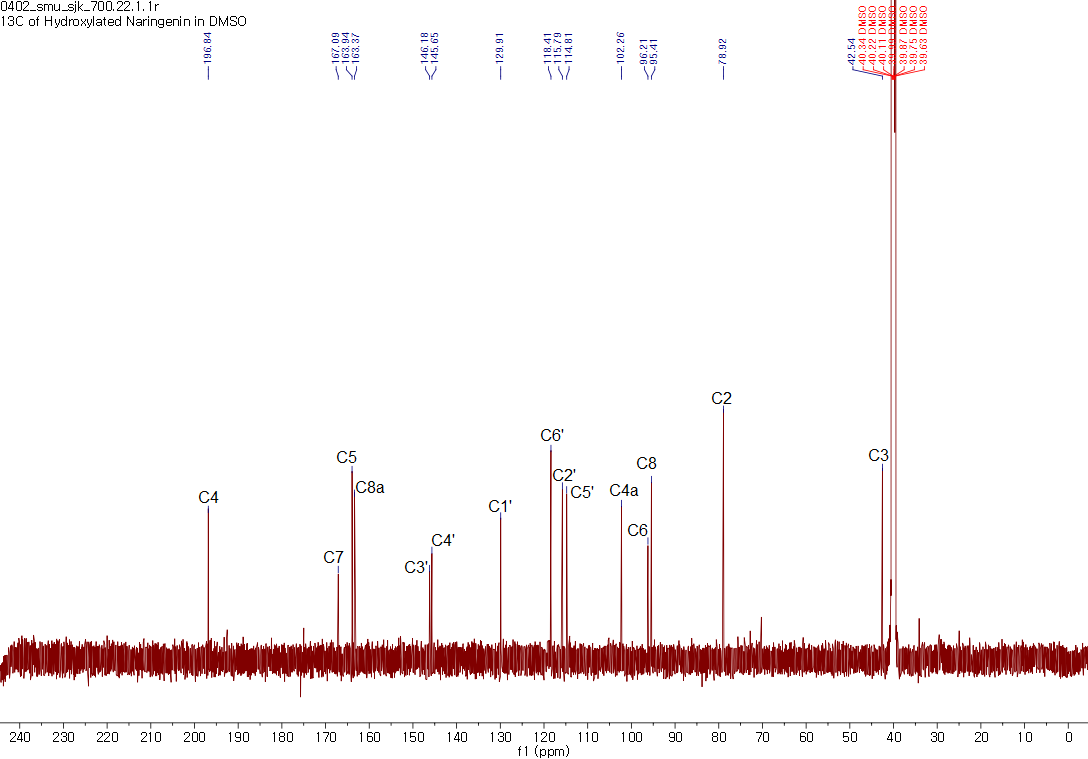

Supplement: Supplementary file 1 — 10.1186/s12934-016-0533-4 Inhibition zone diameter (mm) of four flavonoids along with kanamycin against various Gram-positive and Gram-negative bacteria in disc-diffusion assay. Table S2. IC50 values of four compounds against AGS, HTC116, Hela, HepG2. Figure S1. The UV maxima absorbance and exact mass analysis of naringenin (A) and reaction products P1 (B), P2 (C), P3 (D). P1 have been identified as hydroxylated naringenin while P2 and P3 have been identified as apigenin and hydroxylated apigenin, respectively. Figure S2. HPLC-PDA, the UV maxima absorbance and HR-QTOF ESI/MS analysis of reaction products and apigenin standard. Figure S3. HPLC-PDA, the UV maxima absorbance and HR-QTOF ESI/MS analysis of reaction products and 3-HF standard. Figure S4. HPLC-PDA, the UV maxima absorbance and HR-QTOF ESI/MS analysis of reaction products and flavanone standard. Figure S5. HPLC-PDA, the UV maxima absorbance and HR-QTOF ESI/MS analysis of reaction products and genistein standard. Figure S6. HPLC-PDA, the UV maxima absorbance and HR-QTOF ESI/MS analysis of reaction products and daizein standard. Figure S7. HPLC-PDA, the UV maxima absorbance and HR-QTOF ESI/MS analysis of reaction products and biochanin A standard. Figure S8. 1-Dimensional NMR of naringenin standard. Figure S9. 1-Dimensional NMR of eriodictyol. [file 12934_2016_533_MOESM1_ESM.docx]
